# A new mechanism of interferon’s antiviral action: Induction of autophagy, essential for paramyxovirus replication, is inhibited by the interferon stimulated gene, TDRD7

**DOI:** 10.1371/journal.ppat.1006877

**Published:** 2018-01-30

**Authors:** Gayatri Subramanian, Teodora Kuzmanovic, Ying Zhang, Cara Beate Peter, Manoj Veleeparambil, Ritu Chakravarti, Ganes C. Sen, Saurabh Chattopadhyay

**Affiliations:** 1 Department of Medical Microbiology and Immunology, University of Toledo College of Medicine, Toledo, OH, United States of America; 2 Department of Immunology, Lerner Research Institute, Cleveland, OH, United States of America; 3 Department of Surgery, University of Toledo College of Medicine, Toledo, OH, United States of America; Georgia State University, UNITED STATES

## Abstract

The interferon (IFN) system represents the first line of defense against a wide range of viruses. Virus infection rapidly triggers the transcriptional induction of IFN-β and IFN Stimulated Genes (ISGs), whose protein products act as viral restriction factors by interfering with specific stages of virus life cycle, such as entry, transcription, translation, genome replication, assembly and egress. Here, we report a new mode of action of an ISG, IFN-induced TDRD7 (tudor domain containing 7) inhibited paramyxovirus replication by inhibiting autophagy. TDRD7 was identified as an antiviral gene by a high throughput screen of an ISG shRNA library for blocking IFN’s protective effect against Sendai virus (SeV) replication. The antiviral activity of TDRD7 against SeV, human parainfluenza virus 3 and respiratory syncytial virus was confirmed by its genetic ablation or ectopic expression in several types of mouse and human cells. TDRD7’s antiviral action was mediated by its ability to inhibit autophagy, a cellular catabolic process which was robustly induced by SeV infection and required for its replication. Mechanistic investigation revealed that TDRD7 interfered with the activation of AMP-dependent kinase (AMPK), an enzyme required for initiating autophagy. AMPK activity was required for efficient replication of several paramyxoviruses, as demonstrated by its genetic ablation or inhibition of its activity by TDRD7 or chemical inhibitors. Therefore, our study has identified a new antiviral ISG with a new mode of action.

## Author summary

The antiviral functions of interferons (IFNs) are mediated by the IFN-induced proteins, encoded by the IFN Stimulated Genes (ISGs). Because ISGs are virus-specific, we performed a high throughput genetic screen to identify novel antiviral ISGs against Sendai virus (SeV), a respirovirus of the *Paramyxoviridae* family. Our screen isolated a small subset of anti-SeV ISGs, among which we focused on a novel ISG, Tudor domain containing 7 (TDRD7). The antiviral activity of TDRD7 was confirmed by genetic ablation of the endogenous, and the ectopic expression of the exogenous, TDRD7 in human and mouse cell types. Investigation of the mechanism of antiviral action revealed that TDRD7 inhibited ‘virus-induced autophagy’, which was required for the replication of SeV. Autophagy, a cellular catabolic process, was robustly induced by SeV infection, and was inhibited by TDRD7. TDRD7 interfered with the ‘induction’ step of autophagy by inhibiting the activation of AMP-dependent Kinase (AMPK). AMPK is a multifunctional metabolic kinase, which was activated by SeV infection, and its activity was required for virus replication. Genetic ablation and inhibition of AMPK activity by physiological (TDRD7) or chemical (Compound C) inhibitors strongly attenuated SeV replication. The anti-AMPK activity of TDRD7 was capable of inhibiting other members of *Paramyxoviridae* family, human parainfluenza virus type 3 and respiratory syncytial virus. Therefore, our study uncovered a new antiviral mechanism of IFN by inhibiting the activation of autophagy-inducing kinase AMPK.

## Introduction

Interferon (IFN) system provides the first line of immune defense against viral infections in vertebrates [1–3]. It is designed to inhibit viral infection by blocking virus replication and eliminating the virus-infected cells. The Pattern Recognition Receptors (PRRs), e.g. Toll Like Receptors (TLRs), RIG-I Like Receptors (RLRs) and cyclic AMP-GMP synthase (cGAS)/stimulator of IFN genes (STING), are located in distinct cellular compartments, to sense specific viral components, such as the viral nucleic acids [4–9]. Upon ligand stimulation, the PRRs trigger rapid downstream signaling pathways via respective adaptor proteins to activate the transcription factors, e.g. Interferon Regulatory Factors (IRFs) and Nuclear Factor-κB (NF-κB). The co-operative action of these transcription factors triggers the synthesis of Type-I interferons e.g. IFN-β, an extensively studied antiviral cytokine. After synthesis in the infected cells, IFN-β is secreted and acts on the infected as well as yet uninfected cells via Janus Kinase (JAK)/Signal Transducer of Transcription (STAT) signaling pathways to trigger the synthesis of a number of antiviral genes.

All biological effects of IFN are executed by the induced proteins, encoded by Interferon Stimulated Genes (ISGs), which are either not present or expressed at a low level in untreated cells, but can be transcriptionally upregulated by IFN-action [3, 10, 11]. Most ISGs can also be induced directly in the virus-infected cells without IFN-action [12]. The ISGs perform all physiological and pathological, including viral and non-viral, functions of IFNs. The ISGs function singly or in combination with other ISGs to inhibit virus replication. The antiviral activities of only a handful of these ISGs have so far been identified. Among them, Protein Kinase R (PKR), 2’5’ Oligoadenylate Synthetase (OAS), Mx1, IFN-induced protein with tetratricopeptide repeats (IFIT), tripartite motif (TRIM) family are most well-known for their antiviral activities against a wide spectrum of viruses *in vitro* and *in vivo* [13–20]. PKR, upon binding to viral double-stranded RNA (dsRNA), is activated and phosphorylates eukaryotic initiation factor (eIF2α), leading to the translational inhibition of cellular and viral mRNAs [21]. Mx1 is a broad antiviral ISG that acts at an early stage of virus replication, by sequestering the viral components from the desired destination within the cells [18]. OAS recognizes dsRNA and produces 2’,5’-oligoadenylates, which activate the latent ribonuclease, RNase L that degrades both cellular and viral RNAs [14]. The IFIT family of ISGs recognizes viral mRNAs and thereby inhibiting their translation [17, 19]. IFIT proteins also directly modulate cellular translation machinery by inhibiting eIF3 activities [22]. The TRIM family of proteins, which possesses E3 ubiquitin ligase activity, has diverse cellular functions [20]. In addition to directly interfering with virus life cycle, the ISGs often exert their antiviral actions by amplifying the cellular IFN responses [10]. Many of the ISGs also serve as PRRs or signaling intermediates, which are expressed at low levels and are transcriptionally induced by IFN signaling.

IFN can inhibit many stages of virus replication: viral entry, transcription, replication, translation, assembly or egress. IFN induced transmembrane proteins (IFITM) mediate antiviral resistance to a wide range of viruses [23]. IFITM proteins target the attachment and uncoating, two very early stages of viral entry, of several enveloped viruses [24]. Viperin inhibits Hepatitis C Virus (HCV) replication by localizing to the cellular lipid droplets, the site of viral replication [25]. Viperin also inhibits the budding and release of Influenza A virus by disrupting lipid rafts [26]. Tetherin (BST2) prevents the release of human immunodeficiency virus (HIV-1) by tethering HIV-1 virion particles to the cell surface [27]. In addition to targeting individual steps, multiple ISGs may target different steps of virus replication and elicit a large cumulative antiviral effect [3]. A single ISG can also function in cell type-specific manner to exhibit antiviral defense against multiple viruses. Ifit2 provides protection against a wide range of viruses in specific cells and tissues [17].

Viruses take advantage of cellular machineries or their components to achieve productive replication in the infected cells. Autophagy is an evolutionary conserved cellular degradation pathway, which has numerous physiological functions, including maintenance of cellular homeostasis and host defense [28–30]. Autophagy is induced by various cellular stresses, such as nutrient deprivation or microbial infection. Autophagy generates double-membranous cytoplasmic structure, known as autophagosome, which fuses with the lysosome, leading to the degradation of undesired cellular contents [31]. Adenosine monophosphate (AMP)-dependent Kinase (AMPK) directly phosphorylates the critical Ser/Thr residues of Unc-51 like autophagy activating kinase 1 (ULK1) to initiate the autophagy pathway [32]. Autophagy is regulated by class III PI3 Kinase (PI3K III) and mammalian target of rapamycin (mTOR) [33, 34]. How autophagy regulates virus replication is not clear; however, many viruses utilize autophagy or its components to promote their replication. Autophagy induced by virus infection can have both pro- or anti-viral effects [35–37]. Hepatitis C Virus (HCV), Dengue virus and Poliovirus use autophagosome membranes for their replication [38–40]. Human parainfluenza virus 3 (HPIV3) triggers autophagy in the infected cells and the viral P protein blocks the degradation of autophagosome, to enhance the intracellular virus yield [41]. Measles virus sequesters RIG-I within autophagosome, to evade the antiviral action of IFN [42]. In dendritic cells, Respiratory Syncytial Virus (RSV) induces autophagy, which regulates the adaptive immune responses [43]. How the IFN system regulates ‘virus-induced autophagy’ is unclear.

Paramyxoviruses are strong inducers of IFN and ISGs in the infected cells. To identify the ISGs that can inhibit their replication, we performed a high throughput genetic screen of individual ISGs for their ability to inhibit the replication of Sendai virus (SeV) (family: *Paramyxoviridae*, sub-family: Paramyxovirinae, genus: Respirovirus). Our screen identified a small subset of antiviral ISGs, including Tudor domain containing 7 (TDRD7), which strongly inhibited the replication of SeV in human and mouse cells. The TDRD family of proteins contains multiple Tudor domains and have roles in cellular RNA metabolism. *Tdrd7*^*-/-*^ mice show defects in lens development and spermatogenesis, which are related to the deficiency in Tdrd7-associated mRNAs [44, 45]. Our results demonstrate that TDRD7 inhibits the replication of not only SeV, but also other paramyxoviruses, in multiple cell types. In-depth mechanistic studies revealed that the antiviral effect of TDRD7 is mediated by its ability to inhibit ‘virus-induced autophagy’, which is required for paramyxovirus replication.

## Results

### Identification of anti-SeV ISGs by a high throughput genetic screen

**Fig 1:**
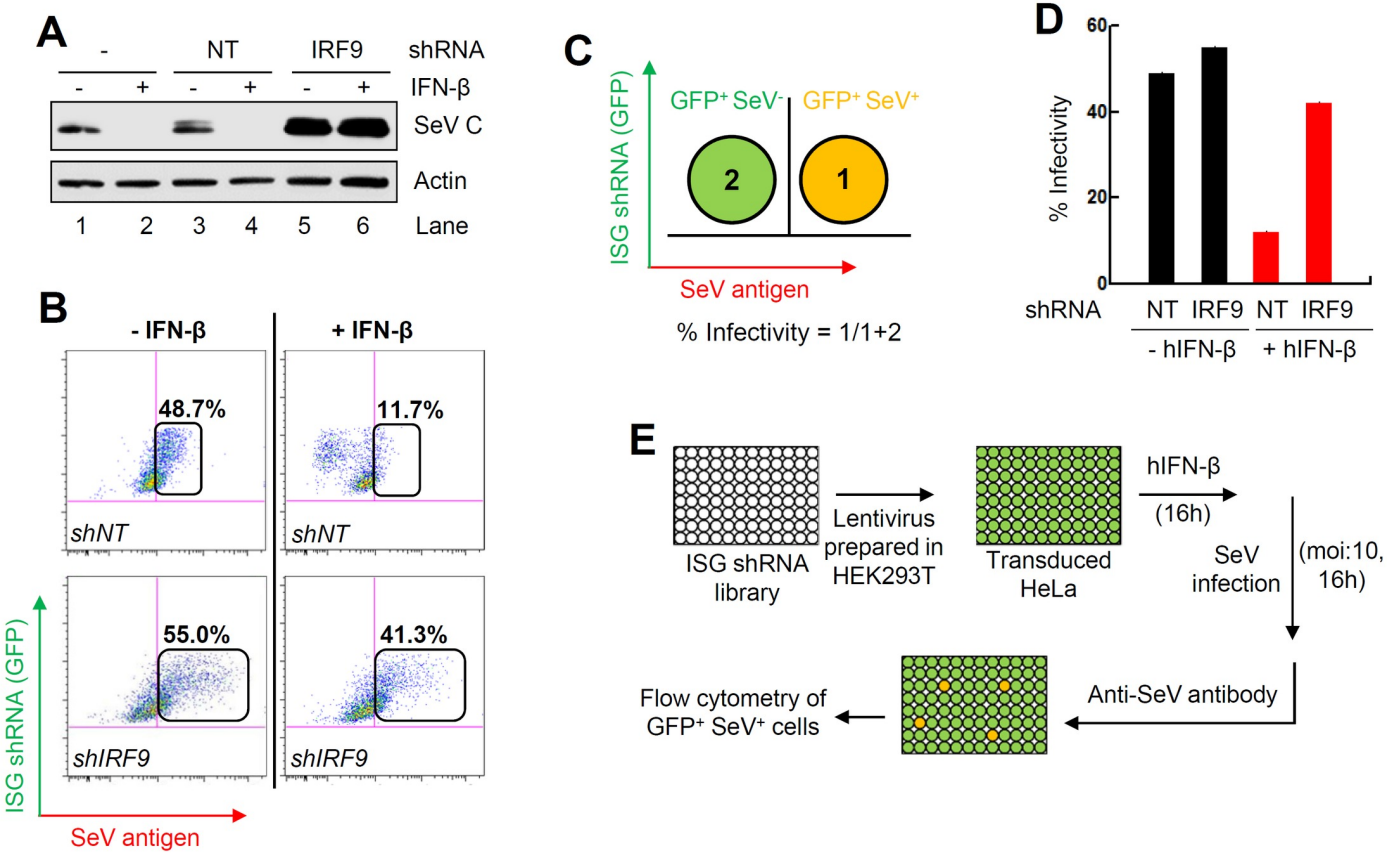
Setting up of high throughput genetic screen of human ISG shRNA library to identify anti-SeV ISGs. **(A)** HeLa cells stably expressing shRNA against IRF9 or a non-targeting (NT) control were pre-treated with human IFN-β for 16 h, when the cells were infected with SeV (moi:10). SeV C protein expression was analyzed by immunoblot at 16 hpi. **(B)** HeLa cells stably expressing shRNA against IRF9 or NT, were pre-treated with IFN-β for 16 h, when the cells were infected with SeV; 16 h later flow cytometric analyses were performed after immunostaining the cells with anti-SeV antibody. **(C)** A strategy to quantify percent SeV infectivity using flow cytometric procedure, as described in (B). The numbers indicate each quadrant and their respective cell population. **(D)** Percent SeV infectivity in NT or IRF9 shRNA-expressing HeLa, infected with SeV in the absence or the presence of IFN-β pre-treatment. **(E)** Our strategy to screen the human ISG shRNA library to isolate anti-SeV ISGs.

**Fig 2:**
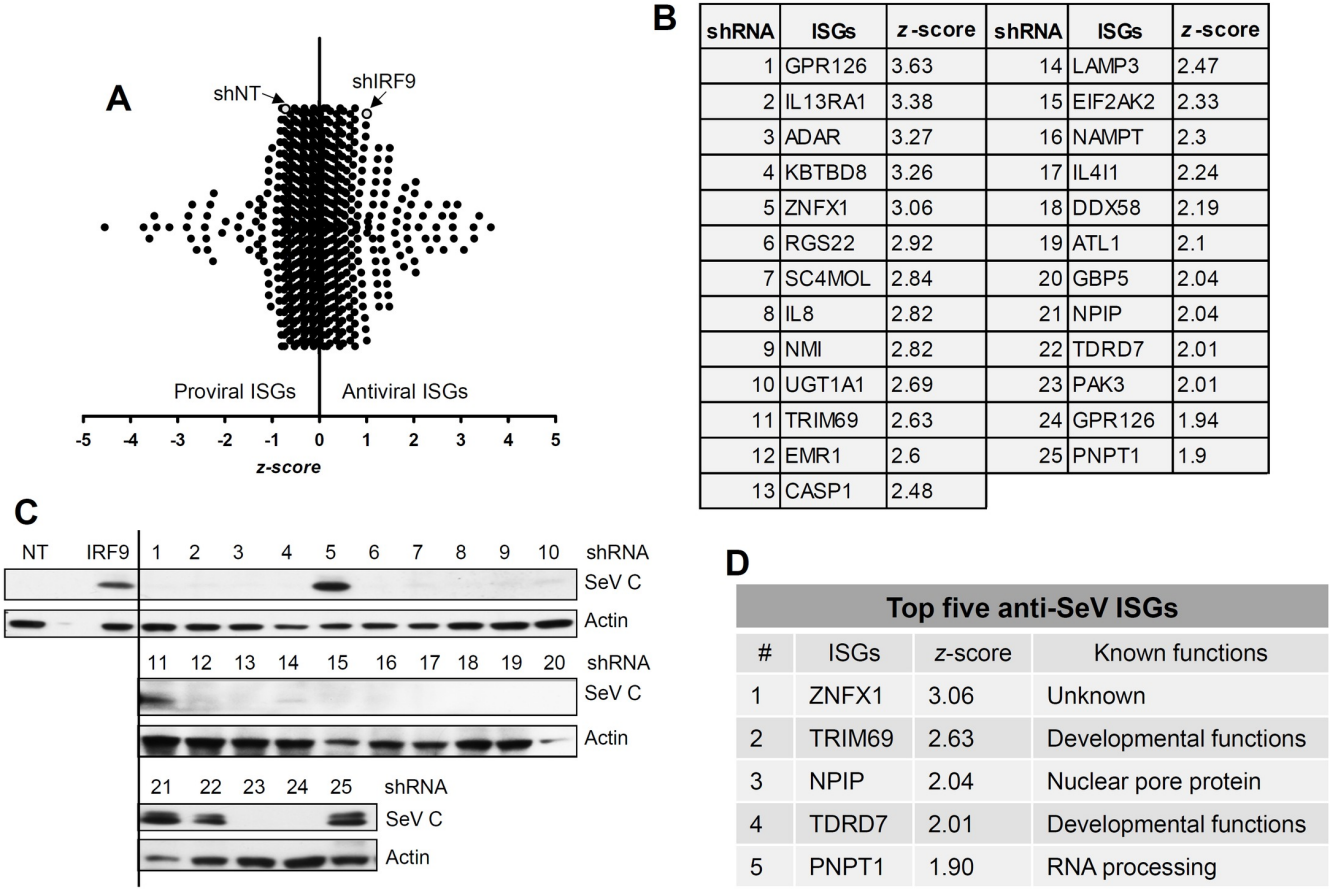
Secondary validation of the primary screen results to identify the most effective anti-SeV ISGs. **(A)** The *z*-scores of all the ISG shRNAs based on their anti-SeV activities; all *z*-scores were normalized to that of shIRF9. **(B)** The *z*-scores of the top 25 primary hits that were used for secondary validation. **(C)** HeLa cells, stably expressing the shRNAs against specific ISGs (indicated above by the numbers, 1–25) were treated with IFN-β for 16 h, when the cells were infected with SeV and analyzed for SeV C protein by immunoblot at 16 hpi. **(D)** The secondary validated anti-SeV ISGs, their *z*-scores and known functions.

To identify the ISGs that block SeV replication, we set up an unbiased genetic screen using a shRNA library against human ISGs (GIPZ lentiviral shRNAmir, Open Biosystems) in HeLa cells. The library is a 96-well formatted commercial system, which packages lentiviruses encoding individual ISG shRNA, a GFP reporter, and a puromycin selection cassette [46]. The GFP expression allowed a flow cytometry-based screen, in which lentivirus-transduced cells were tracked by GFP. The SeV-infected cells were stained with an antibody against the whole virion. In each well of cells, all ISGs were induced by IFN-β-pretreatment but the expression of only one of them was prevented by transduction of the cognate shRNA; the cells were then infected with SeV and the degree of virus replication was measured. Reversal of IFN-mediated inhibition of virus replication in a specific well indicated that the corresponding ISG was responsible for inhibiting SeV replication. We optimized the assay by knocking down the expression of genes required for IFN signaling and, therefore, would exhibit a phenotype. In HeLa cells, the knockdown of IRF9 enhanced the expression of viral protein (SeV C) in comparison to the NT control (Fig 1A, lanes 1, 3, 5). Moreover, IRF9 knockdown cells reversed the IFN-β-mediated inhibition of SeV C protein expression (Fig 1A, lanes 2, 4, 6). We used these cells to develop a flow cytometry-based quantitative assay to measure SeV replication as percent of SeV-positive cells within the GFP-expressing cell population (Fig 1B). The results indicate that knockdown of IRF9 reversed the IFN-β-mediated inhibition of SeV replication (Fig 1B). We quantified the GFP-expressing SeV-antigen positive cells (% infectivity) by flow cytometry (Fig 1C). IRF9 knockdown led to enhanced SeV replication (% infectivity) in both untreated and IFN-β-treated cells (Fig 1D). We screened the shRNA library, which consists of 814 lentiviral constructs (multiple targets for each ISG) expressing shRNA against more than 300 human ISGs. The shRNA library was lentivirally expressed in HeLa cells, using the strategy outlined in Fig 1E. A shRNA against IRF9 was used as an internal control to validate the effect of IFN-β (as in Fig 1D) for each screening experiment. Percent infectivity was quantified for each shRNA (as described in Fig 1C), and was used to calculate *z*-score for the individual shRNAs and normalized to that of IRF9 (Fig 2A and S1 Table) [47]. A set of 25 primary shRNA hits were shortlisted on the basis of high *z*-scores (>1.9, Fig 2B), and used for secondary validation. The primary hits consisted of both known, as well as some novel, antiviral ISGs. In order to further validate these primary hits, stable HeLa cell lines expressing the individual ISG shRNAs were generated. In the ISG shRNA-expressing cells, the reversal of IFN-β-mediated inhibition of SeV C protein expression was measured. As a control, we used IRF9-specific shRNA expressing cells, which alleviated the IFN-β-mediated suppression of SeV C protein level (Fig 2C, left). From the secondary validation assay, we narrowed down to a small subset of five ISGs, the knockdown of which reversed IFN-β-mediated inhibition of SeV C protein expression (Fig 2C and 2D). Importantly, individual knockdown of any of these five ISGs, elevated the level of SeV replication in IFN-β-treated cells (S1 Fig).

### TDRD7 is induced by SeV infection

**Fig 3:**
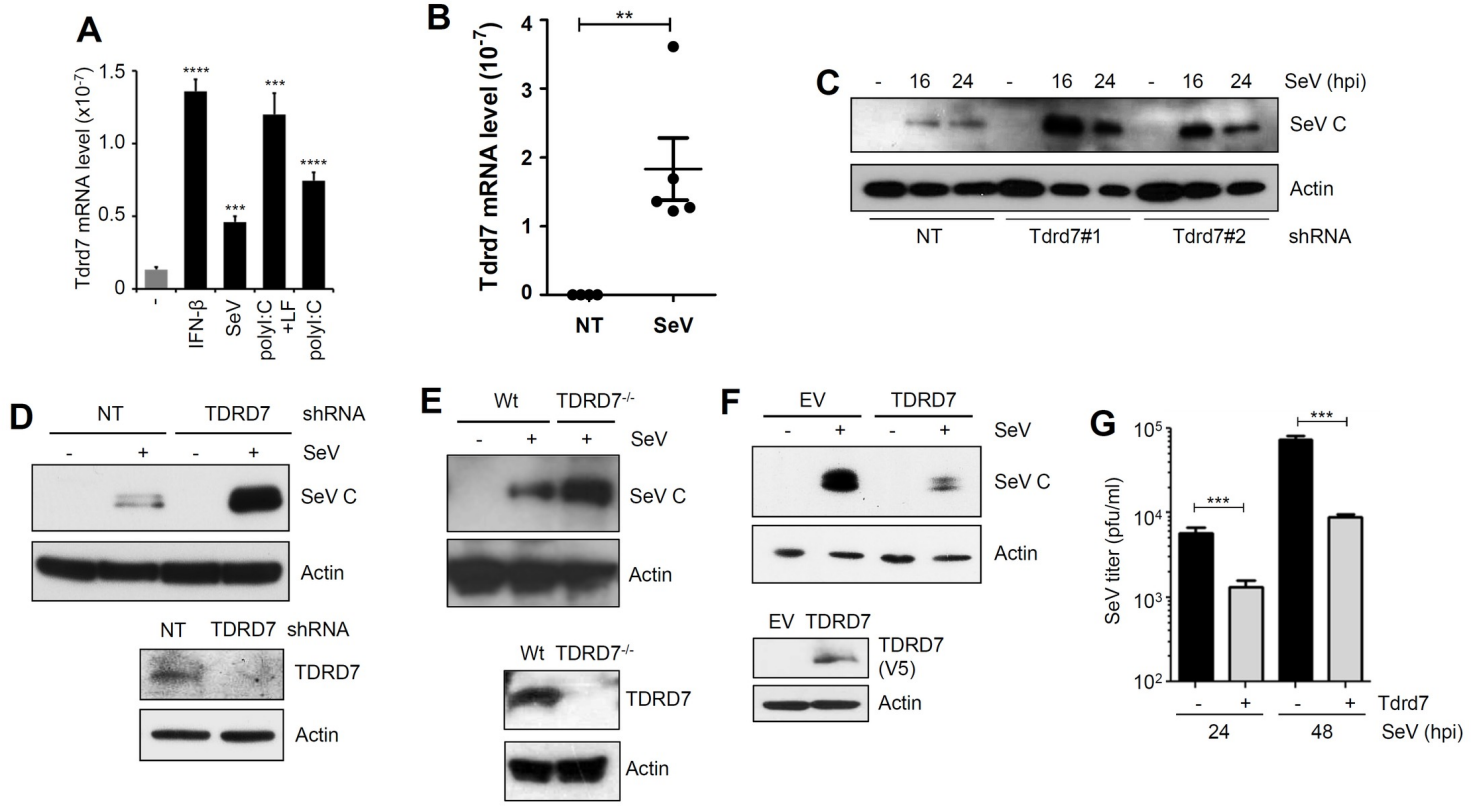
TDRD7 inhibits SeV replication in human and mouse cells. **(A)** QRT-PCR analyses of Tdrd7 induction by various treatments or SeV infection in mouse macrophages (RAW264.7). Treatments: IFN-β: 1000 U/ml, SeV (moi:10), polyI:C+LF: poly(I:C) transfected with Lipofectamine 2000 (LF) or naked poly(I:C). **(B)** QRT-PCR analyses of Tdrd7 mRNA levels in the lungs of mock-infected (NT, PBS-treated) or SeV (52 strain) infected mice after 2 days of infection. **(C)** LA4 cells stably expressing two different Tdrd7 shRNAs (#1 and #2), were infected with SeV for the indicated time, when the SeV C protein levels were analyzed by immunoblot. **(D)** ARPE19 cells stably expressing TDRD7-specific shRNA, were analyzed for SeV C protein expression after SeV infection. Lower panel shows the TDRD7 expression by immunoblot. **(E)** HT1080 (Wt) or TDRD7 knockout (TDRD7^-/-^) cells were infected with SeV and analyzed for SeV C protein expression by immunoblot (upper panel). TDRD7 protein expression is shown in the lower panel by immunoblot. **(F)** HEK293T cells, stably expressing V5.TDRD7 (lower panel), were analyzed for SeV C expression (upper panel) by immunoblot after SeV infection. TDRD7 protein expression is shown in the lower panel by immunoblot. **(G)** L929 cells stably expressing Tdrd7, were analyzed for infectious SeV production at the indicated time post SeV infection. *NT*, *non-targeting*, *EV*, *empty vector*, * indicates p<0.*05*. The results presented here are representatives of at least three biological repeats.

Among the five anti-SeV ISGs identified by our screen, we focused on TDRD7 (in human and Tdrd7 in mouse) because: (a) it is a cytosolic protein and, therefore, is a potential candidate to inhibit paramyxoviruses, which replicate in the cytosol, (b) it has defined functional domains, which may be required for its antiviral action, (c) it has no known functions as an ISG or a viral restriction factor and (d) our previous microarray results indicated its robust transcriptional induction by virus infection [48]. In HeLa cells, our screening system, TDRD7 was transcriptionally induced by IFN treatment (S2A Fig) and the stable knockdown of TDRD7 (S2A Fig) had no impact on the cell viability (S2B Fig). We further examined the inducibility of Tdrd7 in various mouse cells. In RAW264.7 cells, Tdrd7 mRNA was induced, as expected, by IFN-β; it was also induced by adding poly(I:C) to the medium to activate the TLR3 signaling pathway or transfecting poly(I:C) to activate the RLR pathway (Fig 3A). SeV infection, which activates the RLR pathway, induced Tdrd7 mRNA as well (Fig 3A). Similar induction by two strains of SeV was observed in mouse primary bone marrow-derived macrophages (BMDMs) and mouse embryonic fibroblasts (MEFs) (S2C–S2E Fig). The viral induction of Tdrd7 in MEFs was triggered by IRF3-mediated induction of IFN, because it was not observed in *Irf3*^*-/-*^ or *Stat1*^*-/-*^ cells (S2D and S2E Fig). Importantly, Tdrd7 mRNA was strongly induced in SeV-infected mouse lungs (Fig 3B). These results clearly demonstrate that Tdrd7, an ISG, is induced in virus-infected cells and tissues.

### SeV replication is inhibited by Tdrd7

To investigate the antiviral action of TDRD7, we took two approaches: knockdown or knockout of the endogenous TDRD7 gene and ectopic expression of exogenous TDRD7 in multiple human and mouse cell types. In our test cells, we examined the endogenous levels of TDRD7 and as expected, the protein expression of TDRD7 varied between various cell types (S2F Fig). In mouse lung epithelial cells, LA4, the natural target cells of respiratory viruses, knockdown of endogenous Tdrd7 mRNA by two independent shRNAs enhanced the expression of SeV C protein (Fig 3C, S3A Fig). In human retinal epithelial cells, ARPE19, which express relatively higher levels of endogenous TDRD7 compared to LA4, knockdown of TDRD7 (Fig 3D, lower panel) elevated the expression of SeV C protein (Fig 3D). We confirmed these results in mouse fibroblasts, L929, in which the knockdown of endogenous Tdrd7 (S3B Fig) also enhanced SeV C protein expression (S3C Fig). Similar to HeLa, the stable knockdown of Tdrd7 in L929 cells had no impact on the cell viability (S3D Fig). We further examined the antiviral activity of Tdrd7 in non-transformed immortalized MEFs, in which Tdrd7 was transcriptionally induced by IFN-treatment (S3E Fig) and its stable knockdown (S3E Fig) enhanced the SeV C protein expression (S3F Fig). Using CRISPR/Cas9 system, we generated TDRD7 knockout (TDRD7^-/-^) human HT1080 cells (Fig 3E, lower panel and S3G Fig). SeV C protein expression was elevated in TDRD7^-/-^ cells (Fig 3E, upper panel). In the reciprocal strategy, we ectopically expressed TDRD7 (untagged or V5-tagged) in cells that express low levels of endogenous TDRD7, a scenario that mimics IFN-β-induced synthesis of ISGs. TDRD7 was ectopically expressed in HEK293T cells and confocal analyses showed its cytoplasmic distribution in uninfected cells (S3H Fig). In these cells, TDRD7 strongly inhibited viral protein (SeV C) (Fig 3F) and mRNA (SeV P mRNA, S3I Fig) expression. Similarly, ectopic expression of Tdrd7 inhibited viral protein (SeV C) expression in mouse L929 (S3J Fig) and LA4 (S3K Fig) cells. We investigated whether the TDRD7-mediated inhibition of viral mRNA and protein leads to the reduction of infectious virion production. Indeed, the production of infectious virus particles was inhibited by ectopic expression of Tdrd7 in L929 cells (Fig 3G). In subsequent experiments, we investigated the mechanism of anti-SeV action of Tdrd7.

### SeV induces autophagy, which is required for its replication

**Fig 4:**
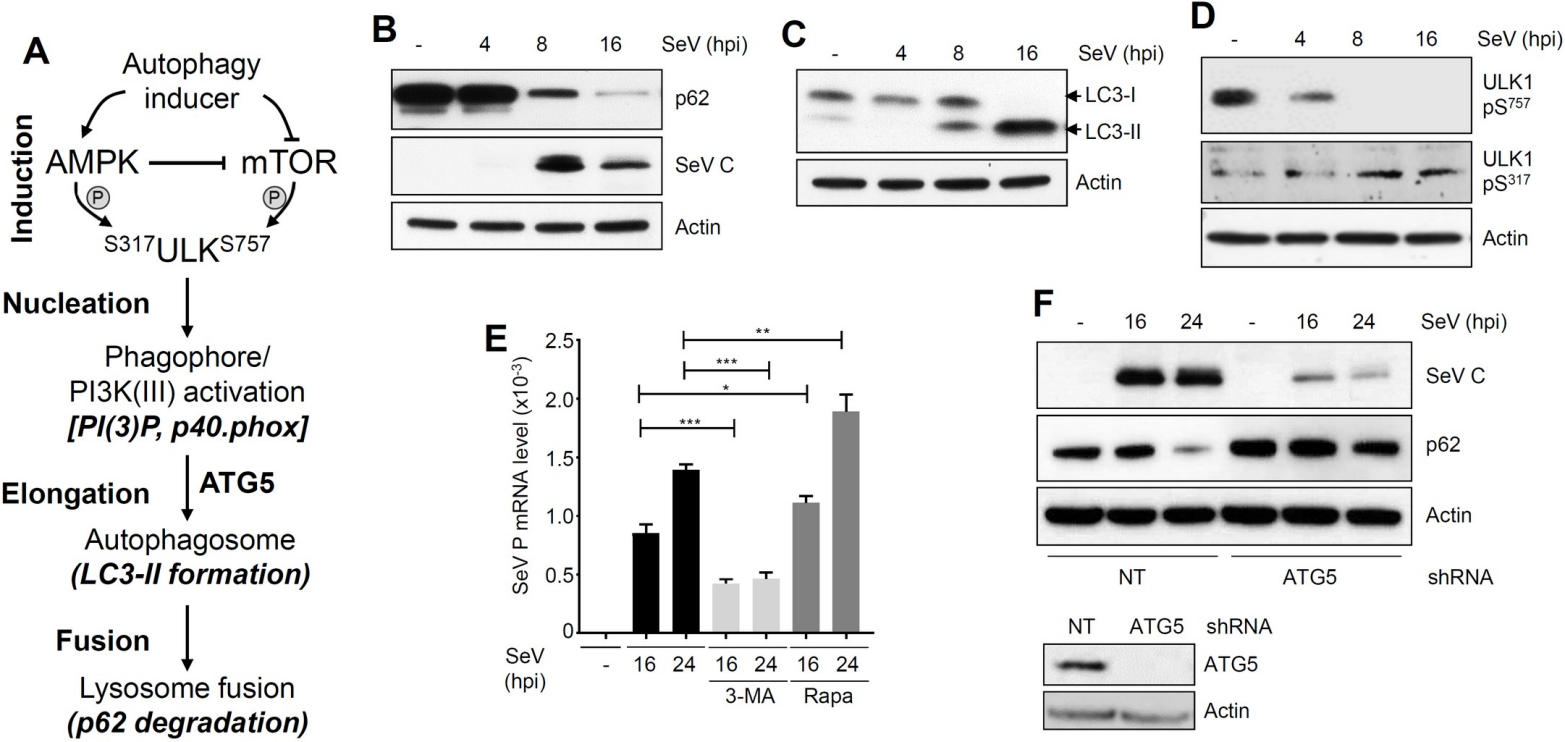
SeV triggers various stages of autophagy to support virus replication. **(A**) Various stages and molecular markers of cellular autophagy. **(B-D)** L929 cells were infected with SeV Cantell (moi: 10) for the indicated time, when the cell lysates were analyzed for p62, SeV C (B), LC3 (C), phospho ULK1 (Ser^757^ or Ser^317^) (D) by immunoblot. **(E)** L929 cells were pre-treated with 3-MA (1 mM) or rapamycin (2 μM) and infected with SeV Cantell (moi:10); SeV P mRNA levels were analyzed by qRT-PCR at the indicated time post infection. **(F)** HT1080 cells, stably expressing ATG5-specific shRNA, were infected with SeV for the indicated time, when SeV C (upper panel) or p62 (middle panel) were analyzed by immunoblot. Lower panel shows the ATG5 levels in these cells by immunoblot. *NT*, *non-targeting*, * indicates p<0.*05*. The results presented here are representatives of at least three biological repeats.

Because the role of autophagy in SeV replication was not clear, we examined various stages of the autophagy pathway (Fig 4A) in SeV-infected cells. In L929 cells, SeV infection triggered robust induction of autophagy, which was analyzed by the degradation of p62 (Fig 4B, upper panel) and the generation of LC3-II (Fig 4C), the indicators of two late stages of the autophagy pathway. The induction of autophagy was correlated with the expression of viral protein (SeV C, Fig 4B, middle panel). In another cell type (LA4), SeV infection similarly triggered autophagy pathway, which was examined by LC3-II generation and p62 degradation (S4A Fig). The molecular signatures of the early stages of autophagy, the dephosphorylation of Ser^757^ and phosphorylation of Ser^317^ of ULK1, were also detected in SeV-infected cells (Fig 4D). These results clearly indicate that SeV infection triggers different stages of the autophagy pathway in multiple cell types. To investigate whether ‘virus-induced autophagy’ is required for SeV replication, we took pharmacological and genetic approaches. A chemical inhibitor of autophagy, 3-MA, inhibited, whereas an activator of autophagy, rapamycin, promoted SeV mRNA synthesis (Fig 4E). Furthermore, chemical inhibitors of various stages of autophagy also significantly suppressed the expression of SeV C protein (S4B Fig). Similarly, knockdown of ATG5 (Fig 4F, lower panel), a key component of autophagy pathway, significantly reduced viral protein expression in human cells (SeV C, Fig 4F). As expected, the SeV-induced autophagy, examined by p62 degradation, was inhibited in these cells (Fig 4F, p62 levels). Collectively, our results clearly indicate that SeV-induced autophagy pathway is required for its replication.

### TDRD7 inhibits autophagy

**Fig 5:**
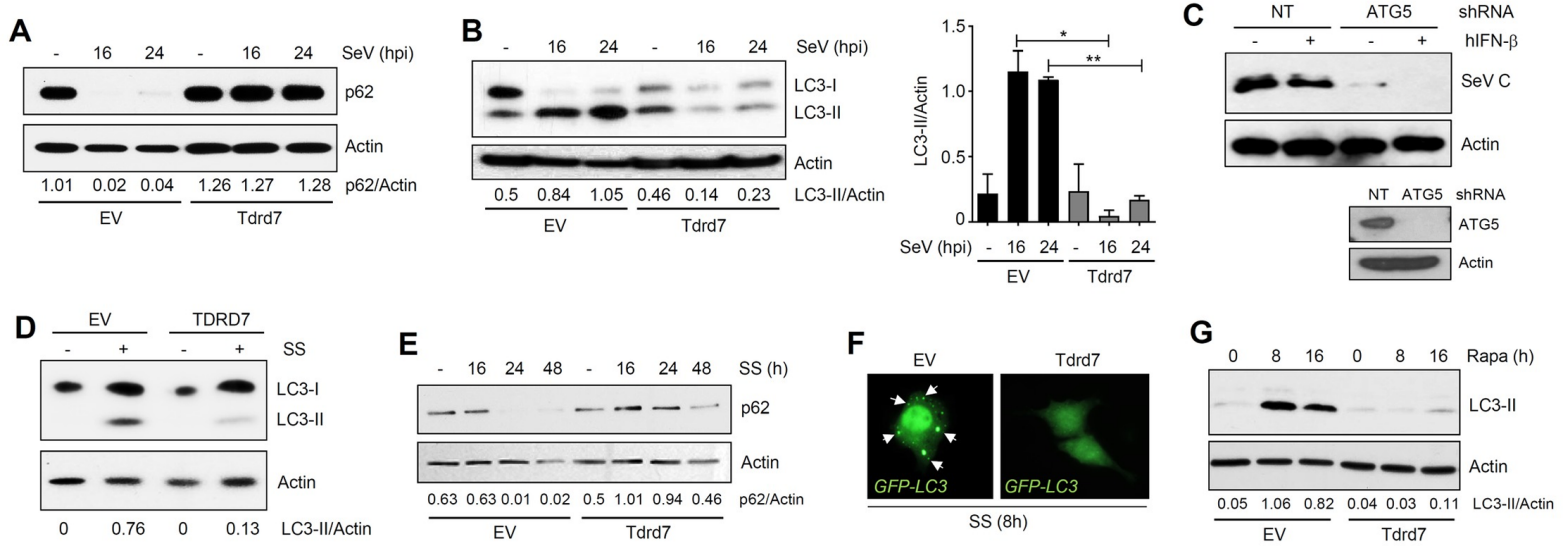
TDRD7 inhibits autophagy induced by viral and non-viral stimuli, to control SeV replication. **(A, B)** L929 cells, stably expressing V5.Tdrd7, were infected with SeV Cantell (moi:10), and analyzed for p62 (A) and LC3 (B) by immunoblot. LC3-II/Actin levels were quantified by Image J. **(C)** TDRD7^-/-^ human cells expressing ATG5-specific shRNA were left untreated or treated with hIFN-β for 16 h, when the cells were infected with SeV and SeV C protein expression was analyzed by immunoblot. ATG5 protein expression is shown in lower panel by immunoblot. **(D)** HEK293T cells stably expressing V5.TDRD7 were serum-starved (SS) and LC3 levels were analyzed after 16 h by immunoblot. LC3-II/Actin ratio are indicated below the Actin panel. **(E)** L929 cells stably expressing V5.Tdrd7 were serum-starved (SS) for the indicated time, when p62 levels were analyzed by immunoblot. **(F)** L929 cells stably expressing V5.Tdrd7 were transfected with GFP-LC3 and serum-starved (SS) for 8 h, when the cells were fixed and analyzed by confocal microscopy. The cytoplasmic puncta structures are shown by arrows. **(G)** L929 cells stably expressing V5.Tdrd7 were treated with Rapamycin (Rapa) for the indicated time, when LC3-II levels were analyzed by immunoblot. *NT*, *non-targeting*, *EV*, *empty vector*, * indicates p<0.*05*. The results presented here are representatives of at least three biological repeats.

The above results led to the hypothesis that TDRD7 might interfere with ‘virus-induced autophagy’ to inhibit SeV replication. To test this, we examined various stages of SeV-induced autophagy in cells, in which TDRD7 expression had been modulated either by ectopically expressing exogenous TDRD7 or by ablating the endogenous TDRD7 levels. In L929 cells, ectopic expression of Tdrd7 strongly inhibited the degradation of p62 (Fig 5A), and the accumulation of LC3-II (Fig 5B). As expected, the knockdown of endogenous TDRD7 in ARPE19 cells, triggered increased accumulation of LC3-II (S4C Fig). Similar results were also obtained in Tdrd7-ablated murine macrophages (RAW264.7) (S4D and S4E Fig). In these cells, Tdrd7 knockdown also elevated SeV C expression (S4E Fig, middle panel). We further investigated whether the antiviral action of TDRD7 is mediated by direct inhibition of autophagy or its ability to modulate IFN and ISG induction. The inhibition of autophagy pathway by knockdown of ATG5 in TDRD7^-/-^ cells suppressed SeV C protein expression (Fig 5C). As expected, these cells restored the ability of IFN to inhibit SeV C expression (Fig 5C). Furthermore, Tdrd7 knockdown cells did not exhibit any significant difference in induction of IFN by SeV (S4F Fig) and ISG (Ifit1) by IFN (S4G Fig). These results demonstrated that the antiviral ISG, TDRD7, inhibits ‘virus-induced autophagy’ to control SeV replication.

Because autophagy is required for normal cellular homeostasis, it is also induced in response to many cellular stresses, other than virus infection. In the next series of experiments, we examined whether TDRD7 could inhibit autophagy induced by nutrient deprivation (e.g. serum starvation, SS and Hank’s Balanced Salt Solution, HBSS) or rapamycin, known activators of the autophagy pathway. As indicated by the LC3-II level, SS-induced autophagy was inhibited by ectopic expression of TDRD7 in human HEK293T cells (Fig 5D). Similarly, in mouse L929 cells, ectopic expression of Tdrd7 inhibited degradation of p62 (Fig 5E) and enhancement of LC3-II levels (S5A Fig). Accumulated LC3-II produces cytoplasmic puncta, which we analyzed by expressing a GFP-LC3 fusion protein in L929 cells. The cytoplasmic puncta formation by LC3-II was significantly reduced in Tdrd7-expressing cells (Fig 5F). Similarly, rapamycin-induced LC3-II formation in L929 cells was inhibited by Tdrd7 (Fig 5G). In Tdrd7-knockdown RAW264.7 cells, rapamycin induced a faster degradation of p62 (S5B Fig) and increased accumulation of LC3-II (S5C Fig). These results demonstrated that Tdrd7 can inhibit autophagy, induced by both viral and non-viral agents, in various human and mouse cell types.

### TDRD7 inhibits an early stage of the autophagy pathway

**Fig 6:**
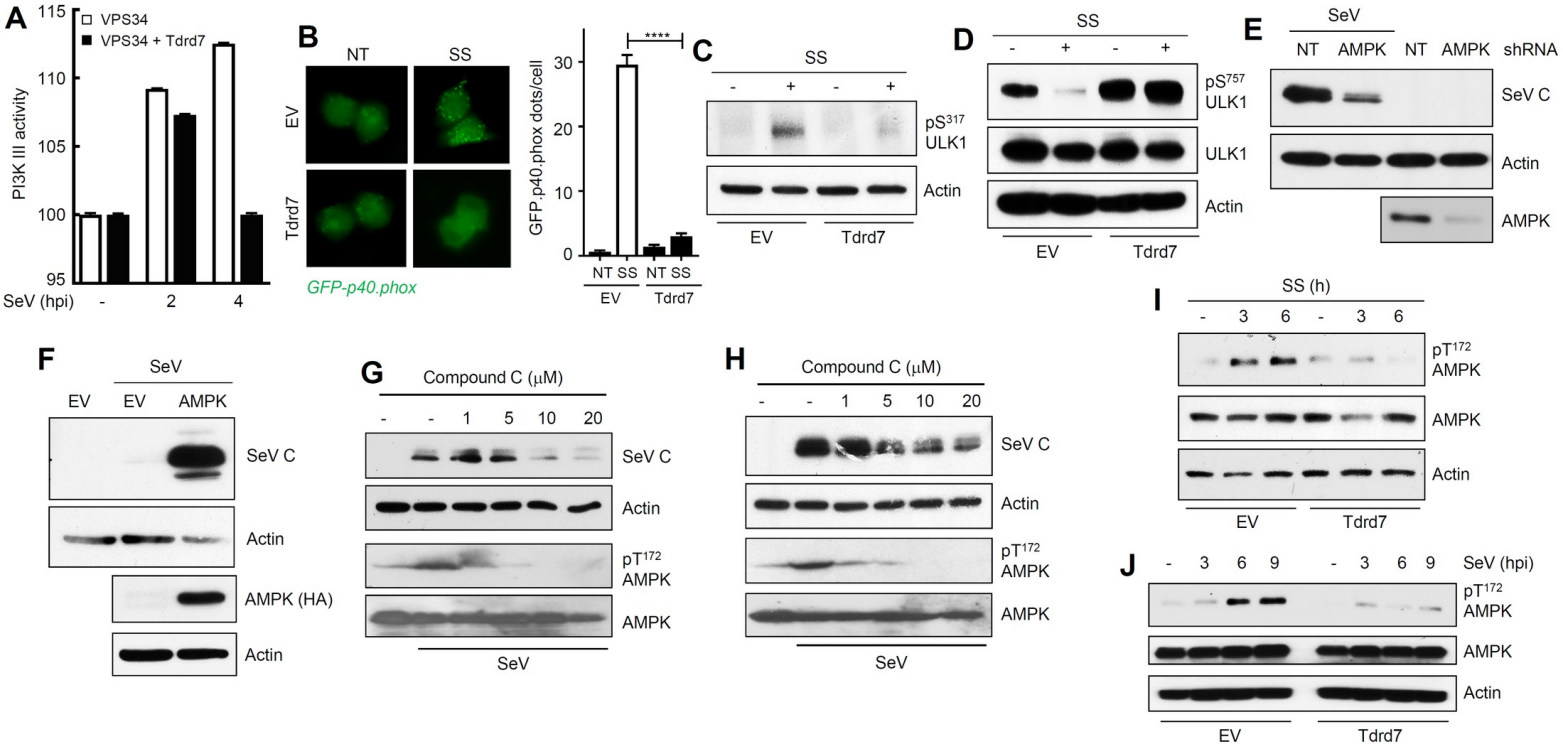
TDRD7 inhibits autophagy-inducing kinase AMPK, whose activity is required for SeV replication. **(A)** L929 cells, transfected with Flag.VPS34 and V5.Tdrd7, were infected with SeV for the indicated time, when VPS34 was immunoprecipitated from the cell lysates and PI3K III activity was analyzed as described in Materials and Methods. **(B)** L929 cells stably expressing V5.Tdrd7 were transfected with GFP.p40.phox and then serum-starved (SS) for 8 h, when the cells were fixed and analyzed by confocal microscopy. The GFP.p40.phox puncta were counted from at least 100 cells and the results are presented on the right panel. **(C)** L929 cells stably expressing V5.Tdrd7 were serum-starved for 8 h, when pULK1 (Ser^317^) was analyzed by immunoblot. **(D)** L929 cells stably expressing V5.Tdrd7 were serum-starved for 8h, when pULK1 (Ser^757^) was analyzed by immunoblot. **(E)** HeLa cells expressing AMPK shRNA were infected with SeV and viral protein (SeV C) expression was analyzed by immunoblot at 16 hpi. Lower panel indicates the AMPK levels in these cells. **(F)** L929 cells ectopically expressing HA-AMPK (lower panel) were infected with SeV and viral protein expression was analyzed at 16 hpi by immunoblot. **(G)** L929 cells were pre-treated with various concentrations of Compound C for 1h, and then infected with SeV. Viral protein (SeV C) expression and pAMPK (on Thr^172^) and AMPK levels were analyzed at 16 hpi by immunoblot. **(H)** HeLa cells were pre-treated with various concentrations of Compound C for 1h, and then infected with SeV. Viral protein (SeV C) expression and pAMPK (on Thr^172^) and AMPK levels were analyzed at 16 hpi by immunoblot. **(I)** L929 cells stably expressing V5.Tdrd7 were serum-starved (SS) for the indicated time, when pAMPK (Thr^172^) levels were analyzed by immunoblot. **(J)** L929 cells stably expressing V5.Tdrd7 were infected with SeV for the indicated time, when pAMPK (Thr^172^) levels were analyzed by immunoblot. *EV*, *empty vector*, *NT*, *no treatment*, * indicates p<0.05. The results presented here are representatives of at least three biological repeats.

Next, to identify the specific target of Tdrd7, we biochemically analyzed the four stages of the autophagy pathway (Fig 4A); for this purpose we used SS of L929 cells to induce autophagy. Because we already knew that the ‘fusion’ (p62 degradation) and the ‘elongation’ (LC3-II levels) steps of the autophagy pathway were inhibited by Tdrd7, we focused our attention to the further upstream pre-elongation steps of the autophagy pathway. As a readout of the ‘nucleation’ step, we analyzed PI3 kinase III activity by direct measurement of PI(3)P produced in virus-infected cells and by the generation of NADPH Oxidase (phox) [49, 50]. In SeV-infected L929 cells, PI3K III was rapidly activated and its activity was inhibited by Tdrd7 expression (Fig 6A, S5D Fig). We further expressed a GFP-conjugated p40 subunit of phox in L929 cells, which produced puncta structures upon autophagy stimulation [49]. Ectopic expression of Tdrd7 decreased the number of phox puncta structures (Fig 6B, left panel), which was quantified by counting the number of puncta structures per GFP-expressing cell (Fig 6B, right panel). These results established that the Tdrd7-mediated block is at the ‘nucleation’ step of the autophagy pathway or further upstream. We examined the effects of TDRD7 on the activation of ULK1, a kinase essential for triggering of the induction stage. ULK1 is activated by phosphorylation of multiple Ser/Thr residues, among which Ser^317^ is a critical residue that is directly phosphorylated by the upstream kinase AMPK [51]. SS triggered robust phosphorylation of ULK1 on Ser^317^, which was inhibited by ectopic Tdrd7 expression (Fig 6C). Activated AMPK inhibits the activity of mTOR, another Ser/Thr kinase, which phosphorylates ULK1 on Ser^757^ to inhibit the autophagy pathway [51]. Therefore, dephosphoryation of Ser^757^ of ULK1 is a positive trigger of the autophagy pathway [51]. SS-induced dephosphorylation of ULK1 (Ser^757^) was also strongly inhibited by Tdrd7 (Fig 6D).

These results pointed to AMPK as the target of TDRD7. AMPK was required for SeV replication: in HeLa cells, knockdown of endogenous AMPK suppressed the expression of SeV C protein (Fig 6E) and ectopic expression of AMPK strongly enhanced SeV C protein expression in L929 cells (Fig 6F). To determine whether only the physical presence or the kinase activity of AMPK is required for virus replication, we used a small molecule chemical inhibitor of AMPK kinase activity, Compound C (CC). Treatment of cells with CC inhibited SeV C protein expression in both human and mouse cells (Fig 6G and 6H), demonstrating that AMPK enzyme activity is required for SeV replication. As expected, CC treatment inhibited phosphorylation of AMPK (on Thr^172^) upon SeV infection (Fig 6G and 6H). We investigated the effect of Tdrd7 on the activation of AMPK, by monitoring the phosphorylation of its Thr^172^. AMPK was rapidly phosphorylated by SS of cells, but Tdrd7 expression inhibited this phosphorylation (Fig 6I). Similarly, HBSS-induced phosphorylation of AMPK on Thr^172^ was also inhibited by Tdrd7 (S5E Fig). Importantly, SeV infection strongly activated AMPK and this step was inhibited by Tdrd7 (Fig 6J). Together, our results clearly demonstrated that Tdrd7 is an inhibitor of the autophagy-inducing kinase AMPK and the antiviral action of Tdrd7 is mediated by its ability to inhibit AMPK activation.

### TDRD7 inhibits the replication of other paramyxoviruses but promotes EMCV replication by the same mechanism

**Fig 7:**
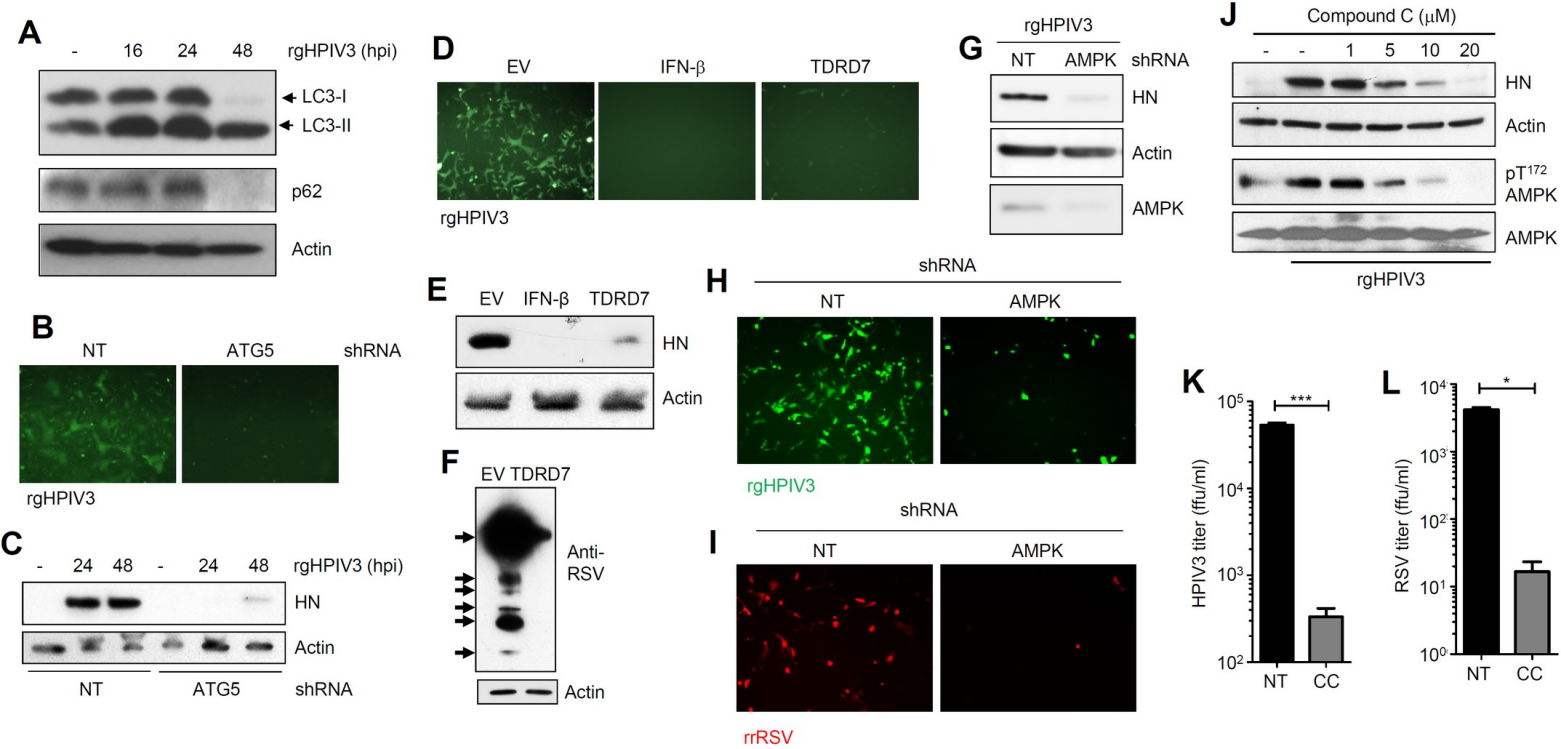
TDRD7 inhibits HPIV3 and RSV replication by anti-AMPK activity. **(A)** HeLa cells were infected with rgHPIV3 (moi:1) and analyzed for LC3 and p62 by immunoblot. **(B, C)** HT1080 cells stably expressing shRNA against ATG5 were infected with rgHPIV3 (moi:1); GFP at 24 hpi (B) and viral HN protein (C) expression were analyzed at the indicated time. **(D, E)** HeLa cells expressing V5.TDRD7 were infected with rgHPIV3 (moi:1), GFP (D) and viral HN protein (E) expression were analyzed at 24 hpi. IFN-β pre-treatment was used as a positive control. **(F)** HeLa cells expressing V5.TDRD7 were infected with RSV (moi:1) and viral protein expression was analyzed at 48 hpi. Arrows indicate the polyclonal serum detecting various viral proteins. **(G)** HeLa cells expressing AMPK shRNA were infected with rgHPIV3 (moi:1) and viral protein (HN) expression was analyzed by immunoblot at 24 hpi. Lower panel indicates the AMPK levels in these cells. **(H)** HeLa cells expressing AMPK shRNA were infected with rgHPIV3 (moi:1) and GFP expression was analyzed by fluorescence microscopy at 24 hpi. **(I)** HeLa cells expressing AMPK shRNA were infected with rrRSV and virus-encoded red fluorescent protein expression was analyzed by fluorescence microscopy at 24 hpi. **(J)** HeLa cells were pre-treated with various concentrations of Compound C for 1h, and then infected with rgHPIV3. Viral protein (HN) and pAMPK (Thr^172^) levels were analyzed at 24 hpi by immunoblot. **(K, L)** HeLa cells were pre-treated with Compound C (CC, 10 μM) for 1h, and then infected with rgHPIV3 (K) or rrRSV (L). Infectious virus particle release in the culture supernatants was analyzed by fluorescence focus assay (expressed in ffu/ml). *NT*, *non-targeting*, *EV*, *empty vector*, * indicates p<0.05. The results presented here are representatives of at least three biological repeats.

Next, we investigated whether TDRD7 can inhibit the replication of other paramyxoviruses by inhibiting autophagy. We chose two clinically important human paramyxoviruses, HPIV3 (family: *Paramyxoviridae*, sub-family: Paramyxovirinae, genus: Respirovirus) and RSV (family: *Paramyxoviridae*, sub-family: Pneumovirinae, genus: Pneumovirus), to examine the generality of TDRD7 action. HPIV3 infection triggered robust autophagy in the infected cells, as examined by the increased LC3-II levels and p62 degradation (Fig 7A). In ATG5 knockdown cells, HPIV3 replication was strongly inhibited, as manifested by the expression of virus-encoded GFP (Fig 7B) and the viral structural protein, HN (Fig 7C). Similar to SeV and HPIV3, RSV infection also triggered autophagy (S6A Fig) and its replication was inhibited in ATG5-knockdown human cells (S6B Fig). To investigate whether TDRD7 inhibits the replication of HPIV3 and RSV, we used TDRD7-expressing human cells. Ectopic expression of TDRD7 inhibited HPIV3 replication, which was examined by both GFP and viral HN expression (Fig 7D and 7E); IFN-β-treatment, a known inhibitor of HPIV3 replication, was used as a positive control. Similar to HPIV3, ectopic expression of TDRD7 strongly inhibited RSV replication, examined by the expression of viral proteins in the infected cells (Fig 7F). As anticipated, AMPK was required for the replication of both HPIV3 and RSV. AMPK-knockdown cells expressed reduced levels of HPIV3-encoded viral protein (HN) (Fig 7G) and GFP (Fig 7H). They also exhibited reduced RSV replication, as indicated by virus-encoded red fluorescent protein expression (Fig 7I). Similar to SeV, HPIV3 infection caused phosphorylation of AMPK and as expected, this was inhibited by CC treatment (Fig 7J). The treatment with CC caused strong reduction of HPIV3 HN protein expression (Fig 7J). In addition to the inhibition of viral protein expression, CC strongly inhibited the production of infectious HPIV3 particles (Fig 7K) and infectious RSV particles (Fig 7L). Our results clearly demonstrated that the kinase activity of AMPK was required for the replication of paramyxoviruses and TDRD7 inhibited their replication by inhibiting AMPK activation. Finally, to examine the specificity of TDRD7 action, we chose a member of another virus family, encephalomyocarditis virus (EMCV; family: *Picornaviridae*, genus: Cardiovirus). EMCV replication, analyzed by the expression of viral RNA polymerase (3DPol), was not inhibited but enhanced by the ectopic expression of TDRD7 (S7 Fig). These results clearly established the specificity of antiviral action of TDRD7 against viruses from different families.

## Discussion

**Fig 8:**
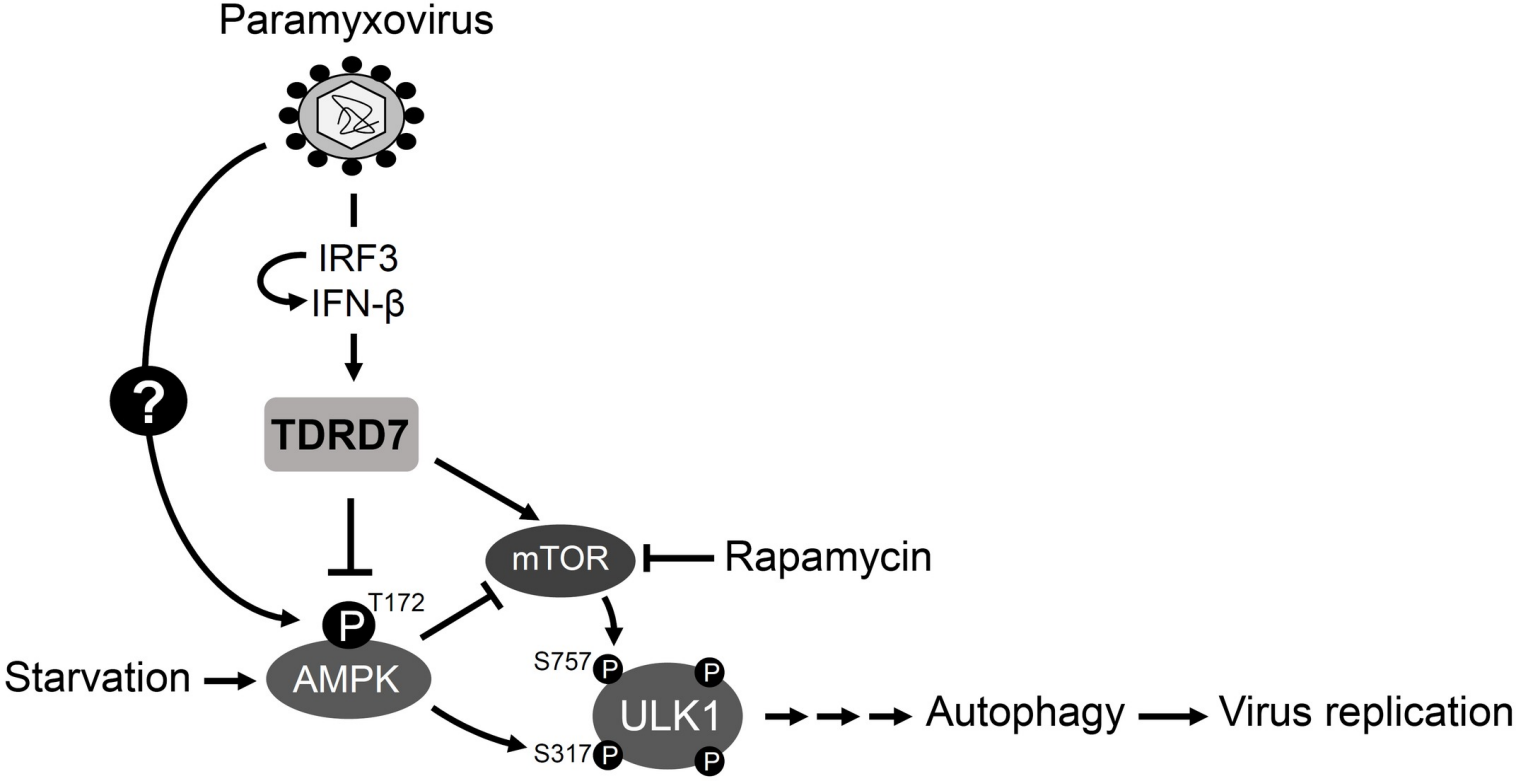
A newly identified antiviral ISG, TDRD7 inhibits paramyxovirus-induced autophagy to control virus replication. The model shows a new mode of action of an ISG, IFN-induced TDRD7 to control paramyxovirus replication by inhibiting cellular autophagy pathway. Paramyxoviruses trigger cellular autophagy by activating the autophagy-inducing kinase, AMPK, by phosphorylation on Thr^172^. AMPK directly phosphorylates ULK1 on Ser^317^ to activate autophagy pathway. Activated AMPK also inhibits mTOR, which phosphorylates ULK1 on Ser^757^ to inhibit autophagy. The newly identified antiviral ISG, TDRD7 inhibits virus-induced autophagy by inhibiting the activation of AMPK, to suppress paramyxovirus replication. Autophagy, induced by nutrient starvation or rapamycin, is also inhibited by TDRD7. Therefore, autophagy inhibition is a new mechanism of the IFN system to control virus replication.

Here, we report a novel mechanism by which the interferon system provides an antiviral response against paramyxoviruses (Fig 8). Using a high throughput genetic screen, we have identified a viral restriction factor, TDRD7, which inhibited paramyxovirus-induced autophagy, a critical step of viral life cycle. Our mechanistic studies revealed that TDRD7 blocks the activation of AMPK, the enzyme that triggers autophagy. As expected, a chemical inhibitor of AMPK’s downstream activities also restricted the replication of paramyxoviruses. Because TDRD7 is a newly discovered viral restriction factor and its antiviral action is novel, we validated the critical results in multiple human and mouse cell types (S2 Table). We, therefore, present a new mechanism by which the IFN system not only provides antiviral protection, but also controls cellular metabolic activity.

In search for a common cellular mechanism that the paramyxoviruses utilize, we uncovered a role of autophagy, which was robustly induced in the early phase of viral life cycle and was required for a stage prior to the transcription of viral mRNA. As a model paramyxovirus, we used SeV, also known as mouse parainfluenza virus type I, because of its wide range of infectivity *in vitro*. SeV triggered a pro-viral autophagy pathway in the infected cells to facilitate its replication. Chemical inhibitors of autophagy blocked, whereas an activator of autophagy promoted, SeV replication. Genetic deficiency of the autophagy pathway significantly inhibited SeV replication. Many RNA viruses use the autophagy pathway to promote their replication [38–42]. Hepatitis C Virus (HCV), Dengue virus and Poliovirus directly use the autophagosome membranes to facilitate their replication [38–40]. Measles virus triggers autophagy to sequester RIG-I in the autophagosome, to inhibit the antiviral action of IFN [42]. In contrast, autophagy impairs the replication of some DNA viruses. In neurons, autophagy is considered an antiviral response against Herpes Simplex Virus (HSV-1) replication [52]. HSV-1 neurovirulence factor, ICP34.5 inhibits autophagy to support virus replication and pathogenesis [53]. However, HSV-2 and Varicella Zoster Virus (VZV), two other α-herpesviruses require basal autophagy to promote their replication [53–56]. The members of γ-herpesviruses, Kaposi’s Sarcoma-associated Herpesvirus (KSHV), Epstein-Barr virus (EBV) antagonize cellular autophagy using viral homologue of Bcl-2 [57]. It will be interesting to investigate whether these viruses can induce TDRD7 and whether TDRD7 has any effect on their replication.

We demonstrated that AMPK, the initiator kinase of the autophagy pathway, is involved in paramyxovirus replication. AMPK was activated by SeV and HPIV3 infection and its activity was required for virus replication. AMPK has multiple cellular functions in addition to initiating autophagy. These include cell growth, mitochondrial biogenesis, and lipid and glucose metabolism [34]. By activating AMPK, the paramyxoviruses may also activate the autophagy-independent activities whose contributions to virus replication remain to be explored. However, our results clearly established that the autophagy-inducing effect of AMPK was critical for virus replication, because ablation of downstream components of the autophagy pathway had similar inhibitory effects. Because paramyxoviruses utilize the autophagy pathway, the role of AMPK in virus replication may be restricted to the activation of autophagy. Whether a specific component of the autophagy pathway, downstream of AMPK, is utilized by the paramyxoviruses, will be investigated in the future. Previous studies have indicated a role of lipophagy and macropinocytosis in promoting AMPK-induced virus replication. Dengue virus promotes AMPK-mTOR signaling pathway to promote lipophagy, a selective autophagy that targets lipid droplets [58]. Vaccinia virus activates AMPK to promote macropinocytosis and actin dynamics, which are required for viral entry into the cells [59]. A kinome screen has revealed that AMPK activity is required for HCMV replication; however, the exact mechanism is currently unknown [60]. KSHV directly interacts with AMPK via its K1 viral protein to promote cell survival [61]. In contrast to these, the picornavirus EMCV replication was promoted in the presence of TDRD7. The exact mechanism behind this will require further investigation.

How the IFN system regulates the autophagy pathway is largely unexplored; however, IFN treatment of cancer cells triggers autophagy via PI3K/mTOR signaling [62]. Some ISGs, e.g. PKR and RNase L promote autophagy in virus-infected cells as antiviral defense mechanisms [63–65]. Several studies indicate crosstalk between autophagy and innate immune signaling pathways; components of autophagy pathway are required for RIG-I signaling [66]. Autophagy is required for TLR7-induced type I IFN production by VSV-infected plasmacytoid dendritic cells [67]. Our study provides a new mechanism of IFN-mediated control of virus replication via inhibition of the autophagy pathway.

Because TDRD7 prevents AMPK activation by non-viral stresses, such as nutrient deprivation, it may have broader effects on AMPK-dependent cellular processes in uninfected cells. For example, IFN is expressed in a low amount in uninfected immune cells, e.g. dendritic cells. The development and biological functions of these cells may be regulated by TDRD7’s anti-AMPK activity. Autophagy is required for cellular homeostasis and unregulated autophagy may lead to disease conditions [68]. In these scenarios, TDRD7-mediated autophagy inhibition would be beneficial. IFN signaling has been shown to inhibit AMPK activation [69]; however, the mechanisms or the involved ISGs are unknown. Our results indicate that TDRD7 is one of the executioner ISGs for the anti-AMPK activity of IFN. AMPK is involved in multiple cellular functions and is activated when cellular ATP levels are low, a scenario that mimics virus infection, which requires high metabolic activity of the infected cells. IFN signaling also regulates the activity of mTOR, a kinase that is involved in protein synthesis. AMPK directly inhibits the activity of mTOR by interacting with intermediate proteins [51, 70]. Therefore, the IFN-mediated inhibition of AMPK may further activate mTOR signaling to enhance the synthesis of desired proteins in the virus-infected cells. Because TDRD7 is known to interact with cellular RNAs [44], this activity may be required for its anti-AMPK functions. AMPK can be activated by long non-coding RNA (lncRNA) [71], and it is speculative that sequestration of the lncRNA activator by TDRD7 may give rise to its AMPK-inhibitory action. Future investigation will be required to explore this possibility.

How paramyxoviruses activate AMPK is an interesting question. To determine whether paramyxoviruses directly trigger AMPK activation by its interaction with viral proteins or by activation of upstream signaling pathways, will require additional investigation. Answers to these questions will lead to therapeutic potential by targeting the virus-AMPK interaction. Because temporary AMPK inhibition is not toxic, the chemical inhibitor is a potential candidate for antiviral therapy. However, AMPK is involved in modulating the functions of both innate immune cells, e.g. macrophages, and the adaptive immune cells, e.g. T cells [72, 73]. Therefore, the use of AMPK inhibition as an antiviral strategy will require in-depth investigation of both innate and adaptive immune responses in virus-infected host. Because type I IFN is also involved in regulating the functions of these cells, TDRD7 may also contribute to the regulation of immune cell functions. TDRD7 knockout mice have only been examined for its role in lens and germ cell development [44, 45]. Because TDRD7 is not present in high amount in majority of the cell types, the transcriptional induction by viruses or IFN exposure will uncover its new roles in other cell types as well. Whether the previously identified functions of TDRD7 can also be regulated by the IFN system will require further investigation.

## Materials and methods

### Cells, plasmids and reagents

Human cell lines HeLa, HT1080, ARPE19, HEK293T, and mouse cell lines L929, LA4, MEFs, RAW264.7 were maintained in DMEM containing 10% FBS, penicillin and streptomycin. All cell lines used in this study were maintained in the laboratory. Expression vectors of human and mouse TDRD7/Tdrd7 gene (untagged) were obtained from Origene and sub-cloned into lentiviral vector (pLVX-IRES-puro, V5-tagged). AMPK expression plasmid was obtained from Addgene and was sub-cloned into lentiviral vector (pLVX-IRES-puro, HA-tagged AMPK) and Flag.VPS34 plasmid was obtained from Addgene. Autophagy inhibitors (3-MA, chloroquine, quinacrine, bafilomycin-1) or activators (rapamycin), AMPK inhibitor (Compound C) were obtained from Sigma-Aldrich, human and mouse IFN-β were obtained from R&D, MTT was obtained from Fisher Scientific, and Lipofectamine 2000 was obtained from Thermo Fisher Scientific. The antibodies against the specific proteins were obtained as indicated below: anti-SeV C: raised in the authors’ laboratory [74], anti-whole SeV antibody was a gift from John Nudrud (Case Western Reserve University), anti-HPIV3 HN: Abcam, anti-RSV: Abcam, anti-3DPol: Santa Cruz, anti-TDRD7: Sigma-Aldrich, anti-LC3: Cell Signaling, anti-p62: Fitzgerald, anti-pULK1(Ser757)/anti-pULK1(Ser317)/anti-ULK1: Cell Signaling, anti-pAMPK(Thr172)/anti-AMPK: Cell Signaling, anti-ATG5: Cell Signaling, anti-Actin: Sigma, anti-V5: Thermo Fisher Scientific, anti-Flag: Sigma-Aldrich, anti-Flag-agarose beads: Sigma-Aldrich.

### shRNA library and screening of ISGs against SeV

A custom generated, lentivirus-based shRNAmir library against human ISGs was obtained from Open Biosystems. The seed sequences for shRNA targeting each ISG have been described before [46]. The vector was designed to co-express the shRNA and GFP by a cytomegalovirus (CMV) promoter. The shRNA plasmids were packaged into lentiviral vectors in 96-well plates using the manufacturer’s instructions. HeLa cells were transduced with these lentiviruses for 48 h, when the cells were treated with 1000 U/ml of IFN-β. Twenty four hours later, the cells were infected with SeV (Cantell) at an MOI of 10. After 16 h, cells were harvested, fixed with 1% paraformaldehyde in phosphate-buffered saline (PBS) for 10 min, permeabilized with 0.1% (wt/vol) saponin, and incubated with an anti-SeV polyclonal antibody and an Alexa Fluor 647-conjugated goat anti-rabbit secondary antibody. Cells were analyzed using a BD LSRFortessa flow cytometer (BD Biosciences) and the data were analyzed by FlowJo. Viral infection was determined based on the percentage of SeV-positive cells in shRNA-transduced (GFP) populations (% infectivity, as illustrated in Fig 1C). The relative infectivity in each well was normalized to the wells containing a shRNA against IRF9 sequence to obtain *z*-scores [47]. Independent lentivirus stocks were used to validate the primary screen results in three independent replicates. Primary hits were defined as those *z* scores greater than 1.9.

### Secondary validation of primary hits

The primary hits obtained from the high throughput screen were validated by stably expressing the respective ISG shRNAs (which were positive in the primary screen) in HeLa cells. We used non-targeting (NT) and IRF9-specific shRNAs as controls. The HeLa cells, stably expressing the shRNAs against the shortlisted ISGs were pre-treated with IFN-β followed by infection with SeV Cantell (moi: 10) and viral protein (SeV C) expression was analyzed by immunoblot.

### Knockdown and ectopic expression

For generating stable knockdown of TDRD7/Tdrd7 genes in human and mouse cells, the respective shRNAs [from the shRNA library, Open Biosystems (TDRD7: GATCGCACATGTTTATTTA, used in all human cells of the study), (Tdrd7#1: CAGGATTTGCCTCAGATTA, used in all mouse cells of the study) or Sigma (Tdrd7#2, SHCLNG-NM_146142, TRCN0000102515, used only in LA4 cells)] were lentivirally expressed and the transduced cells were selected in puromycin containing medium. The stable knockdown cells were evaluated for levels of TDRD7/Tdrd7 by qRT-PCR in the absence or the presence of IFN-treatment or immunoblot. These and the control (NT) cells were evaluated for viral replication. ATG5-specific shRNAs (Sigma # SHCLNG-NM_004849) were stably expressed lentivirally and the transduced cells were selected in puromycin containing medium. AMPK knockdown cells were generated using lentiviral shRNA plasmids (Sigma # SHCLNG-NM_006251) and the transduced cells were selected in puromycin containing medium. Stable cell lines ectopically expressing epitope-tagged human and mouse TDRD7/Tdrd7 using lentiviral delivery systems (pLVX-IRES-puro) and were selected in puromycin containing medium. The stable cells were used for viral infection and other biochemical analyses. Wherever indicated, the stable cell lines were also generated by transfecting the untagged TDRD7/Tdrd7 plasmids (from Origene) and selecting the transfected cells with puromycin. Cells ectopically expressing AMPK were generated by lentivirally transducing HA.AMPK using pLVX-IRES-puro and selecting the cells in puromycin containing medium.

### Generation of CRISPR/Cas9-mediated TDRD7 knockout cells

HT1080 cells were transfected with either control (sc-418922) or TDRD7-specific (sc-407210) CRISPR/Cas9 plasmids. Transfected cells were sorted for high GFP-expressers using flow cytometry, and the GFP-expressing cells were expanded to isolate individual clones. These clones were examined for TDRD7 mRNA levels by qRT-PCR analyses and protein levels by immunoblot.

### Virus infection

SeV Cantell (VR-907) and 52 (VR-105) strains were obtained from Charles River, and the infection procedure has been previously described [74, 75]. Briefly, the cells were infected by the viruses (at an MOI specified in the figure legends) in serum-free DMEM for 1.5 h, after which the cells were washed and replaced with normal growth medium. The virus-infected cells were analyzed at the indicated time for viral protein expression or as described in figure legends. For quantification of infectious SeV particles in the culture medium, standard plaque assays were performed, as described previously [74, 75]. Recombinant RSV (rrRSV) and HPIV3 (rgHPIV3) infections were carried out in serum-free DMEM at the indicated MOI. Infectious rrRSV and rgHPIV3 particles were analyzed by quantification of fluorescent foci forming units or the virus-infected cells were photographed using fluorescence microscope. EMCV infection was carried out using previously published procedure [76], and the infected cells were analyzed by the expression of viral protein, as indicated in the figure legends.

### Analyses of autophagy induction and AMPK activation

For analyses of nutrient starvation induced autophagy, the cells were washed (three times) with and then incubated in serum-free DMEM or HBSS (Lerner Research Institute Cell Culture Core) for the time period indicated in the figure legends. At the end of the incubation period, the cells were harvested and the lysates were analyzed for pAMPK (Thr^172^), pULK1 (Ser^317^ and Ser^757^), LC3, p62. Similarly, for the analysis of puncta formation by GFP-LC3 or GFP.p40.PHOX, the cells were transiently transfected with these plasmids and then serum starvation was carried out. At the end of the exposure, the cells were fixed and confocal microscopy was performed. The puncta structures were manually counted in GFP-expressing cells from multiple fields.

### Cell lysis and immunoblot

Immunoblot was performed using previously described procedures [74, 75]. Briefly, cells were lysed in 50 mM Tris buffer, pH 7.4 containing 150 mM of NaCl, 0.1% Triton X-100, 1 mM sodium orthovanadate, 10 mM of sodium fluoride, 10 mM of β-glycerophosphate, 5 mM sodium pyrophosphate, protease and phosphatase inhibitors (Roche). Total protein extracts were analyzed by SDS-PAGE followed by immunoblot. The density of protein bands on the immunoblots was quantified using Image J program.

### RNA isolation and qRT-PCR analyses

Total RNA was isolated using RNA isolation kit (Roche) and cDNA was prepared using ImProm-II Reverse Transcription Kit (Promega). For qRT-PCR, 0.5 ng of cDNA was analyzed using Applied Biosystem's Power SYBR Green PCR mix in Roche LightCycler. The expression levels of the mRNAs were normalized to 18S rRNA. To investigate *in vivo* gene expression, lungs were harvested from the SeV-infected mice and quickly frozen on dry ice. Total RNA was isolated from frozen lungs using Trizol extraction and the cDNAs were prepared using ImProm-II Reverse Transcription Kit and then subjected to qRT-PCR analyses as described above [74]. For the qRT-PCR analyses of the respective genes, the following primers were used: TDRD7-fwd: CGAGCTGTTCTGCAGTCTCA, TDRD7-rev: GCCATGGCATAGCAGGTAAT, Tdrd7-fwd: CTAAGGGCTGTCCTGCAGTC, Tdrd7-rev: AGAGTTGCCTTTGGCTTT, SeV P-fwd: CAAAAGTGAGGGCGAAGGAGAA, SeV P-rev: CGCCCAGATCCTGAGATACAGA, Ifnb-fwd: CTTCTCCGTCATCTCCATAGGG, Ifnb-rev: CACAGCCCTCTCCATCAACT, Ifit1-fwd: CAGAAGCACACATTGAAGAA, Ifit1-rev: TGTAAGTAGCCAGAGGAAGG, 18S-fwd: ATTGACGGAAGGGCACCACCAG, 18S-rev: CAAATCGCTCCACCAACTAAGAACG.

### Confocal microscopy

HEK293T cells expressing V5.TDRD7 were grown on coverslips, fixed in 4% paraformaldehyde, permeabilized in 0.2% Triton X-100 and subjected to immunostaining by anti-V5 antibody followed by Alexa Fluor-conjugated secondary antibody. The objects were mounted on slides using VectaShield/DAPI and analyzed by confocal microscopy. For GFP.LC3 and GFP.p40.PHOX analyses, the cells expressing these plasmids were analyzed by confocal microscopy. The images were further processed and analyzed using Adobe Photoshop software. Multiple culture fields (at least 100 cells from more than 20 fields) were analyzed to select representative images and for quantification.

### PI3K III activity assay

L929 cells, transfected with Flag.VPS34 and V5.Tdrd7, were infected with SeV for the indicated time (in figure legends), when the cell lysates were immunoprecipitated with Flag-agarose beads. The immunoprecipitates were analyzed for PI3K III activity by measuring the PI(3)P levels using Class III PI3K ELISA kit (Echelon) following manufacturer’s instructions. The PI(3)P levels in the mock-infected VPS34-expressing cells was expressed as 100 and all other values were normalized to this.

### MTT assay to measure cell viability

Cells at the density of 10,000/well were cultured in 96 well plates for 2 days followed by addition of 10μl of 5mg/ml MTT solution in PBS and additional culturing for 4 hours. The water insoluble formazan was dissolved in DMSO and the absorbance was measured at 570nm [77]. The absorbance in control (NT) cells was expressed as 100 and all other values were normalized to this.

### SeV infection in mice

C57BL/6 Wt mice, obtained from Taconic, were either mock-infected (PBS) or intranasally infected with SeV (52 strain, 120,000 pfu), as described previously [74, 78]. The lungs were harvested after 2 days of infection, total RNA was isolated and analyzed by qRT-PCR.

### Statistical analyses

The statistical analyses were performed using GraphPad Prism 5.02 software. The ‘p’ values were calculated using two-tailed, un-paired Student’s t tests and are shown in the relevant figures. The results presented here are the representatives of at least three biological repeats.

## Supporting information

S1 Fig Secondary validation of the anti-SeV ISGs. HeLa cells expressing non-targeting (NT), IRF9 or the ISG-specific shRNA (numbers are indicated in Fig 2B), were left untreated or IFN-β-treated and SeV titers were determined at 24 hpi in the culture supernatants.
(TIF)

S2 Fig The expression and induction of TDRD7 by virus infection. **(A)** Stable knockdown of TDRD7 in IFN-treated HeLa cells were analyzed by qRT-PCR. **(B)** Cell proliferation was analyzed in TDRD7 knockdown HeLa cells by MTT assay. **(C)** Primary BMDMs from Wt C57BL/6 mice were infected with SeV (Cantell and 52 strains) and Tdrd7 mRNA levels were analyzed by qRT-PCR 8 hpi. **(D, E)** MEFs from the indicated genotypes were infected with SeV at moi:10 and Tdrd7 mRNA levels were analyzed by qRT-PCR. **(F)** Endogenous TDRD7 protein expression in various human and mouse cells was analyzed by immunoblot. *NT*, *non-targeting*.
(TIF)

S3 Fig TDRD7 is a newly-identified anti-SeV ISG. **(A)** Stable knockdown of Tdrd7 in LA4 cells by two different shRNAs (#1 and #2) was analyzed by qRT-PCR. **(B)** Stable knockdown of Tdrd7 in L929 cells was analyzed by qRT-PCR. **(C)** L929 cells expressing Tdrd7-specific shRNA were infected with SeV and SeV C levels were analyzed by immunoblot. **(D)** Cell proliferation was analyzed in Tdrd7 knockdown L929 cells by MTT assay. **(E)** Stable knockdown of Tdrd7 in IFN-treated MEFs was analyzed by qRT-PCR. **(F)** MEFs expressing Tdrd7-specific shRNA were infected with SeV and viral protein (SeV C) levels were analyzed by immunoblot. **(G)** Wt and TDRD7^-/-^ HT1080 cells were mock-infected or infected with SeV and TDRD7 mRNA levels were analyzed by qRT-PCR. **(H)** HEK293T cells, stably expressing V5.TDRD7, were immuno-stained with anti-V5 antibody and analyzed by confocal microscopy. **(I)** HEK293T cells, stably expressing V5.TDRD7, were analyzed for SeV P mRNA expression by qRT-PCR after 8h of SeV infection. **(J)** L929 cells ectopically expressing Tdrd7 (Origene, untagged) were infected with SeV and SeV C levels were analyzed by immunoblot. Lower panel indicates the ectopic expression of Tdrd7. **(K)** LA4 cells expressing Tdrd7 (Origene, untagged) were infected with SeV and analyzed for SeV C levels by immunoblot. *NT*, *non-targeting*, *EV*, *empty vector*.
(TIF)

S4 Fig TDRD7 inhibits SeV-induced autophagy, which is required for virus replication, but not IFN or ISG induction. **(A)** LA4 cells infected with SeV (moi:10) for the indicated time, when LC3-II and p62 levels were analyzed by immunoblot. **(B)** HT1080 cells were pre-treated with autophagy inhibitors (Q, quinacrine 10μM, CQ, chloroquine 25μM, Baf, Bafilomycin 100 nM) and infected with SeV for 16h, when SeV C levels were analyzed by immunoblot. **(C)** ARPE19 cells expressing Tdrd7-specific shRNA, were infected with SeV and LC3 levels were analyzed by immunoblot. **(D)** RAW264.7 cells expressing Tdrd7-specific shRNA, were treated with IFN-β and Tdrd7 mRNA levels were analyzed by qRT-PCR. **(E)** RAW264.7 cells expressing Tdrd7-specific shRNA were infected with SeV for the indicated time, when LC3-II and SeV C levels were analyzed by immunoblot. **(F)** *Ifnb* induction was analyzed in Tdrd7 knockdown L929 cells upon SeV infection by qRT-PCR. **(G)** *Ifit1* induction was analyzed in Tdrd7 knockdown L929 cells upon mIFN-β treatment by qRT-PCR. *NT*, *non-targeting*.
(TIF)

S5 Fig Tdrd7 inhibits virus infection and cellular stress-induced autophagy by inhibiting AMPK activation. **(A)** L929 cells stably expressing V5.Tdrd7 were serum-starved (SS) for 16h, when LC3-II levels were analyzed by immunoblot. LC3-II/Actin ratio are shown below the Actin panel. **(B, C)** RAW264.7 cells expressing Tdrd7-specific shRNA were treated with rapamycin (Rapa) for the indicated time when p62 (B) and LC3-II (C) levels were analyzed by immunoblot. **(D)** Expression of Flag.VPS34 and V5.Tdrd7 was analyzed in transfected L929 cells by immunoblot. **(E)** L929 cells stably expressing V5.Tdrd7 were incubated in HBSS for the indicated time, when pAMPK (on Thr^172^) levels were analyzed by immunoblot. *NT*, *non-targeting*, *EV*, *empty vector*.
(TIF)

S6 Fig RSV triggers autophagy to favor virus replication. **(A)** HT1080 cells, infected with rrRSV were analyzed for LC3-II and p62 by immunoblot 24 hpi. **(B)** HT1080 cells expressing non-targeting (NT) or ATG5-specific shRNA, were infected with rrRSV and analyzed for viral protein expression at 48 hpi.
(TIF)

S7 Fig TDRD7 promotes EMCV replication. L929 cells ectopically expressing Tdrd7 were infected with EMCV (moi:1) and viral RNA polymerase (3DPol) expression was analyzed by immunoblot after 8 h. *EV*, *empty vector*.
(TIF)

S1 Table *Z*-scores of ISG shRNAs from the high throughput genetic screen. The percent SeV infectivity for each ISG shRNA (as shown in Fig 1D) was used to calculate the *z*-scores and normalized to that of IRF9 shRNA. The normalized *z*-scores of each of the ISG shRNA in the library are shown in the table.
(PDF)

S2 Table Antiviral activity of TDRD7 in various human and mouse cells. The table summarizes the properties of various human and mouse cell types, used in the study, with respect to their levels of Tdrd7 mRNA and protein expression, antiviral activity and biochemical mechanism. KD, knockdown by lentiviral transduction of shRNA plasmids, KO, knockout by CRISPR/Cas9, ectopic expression by lentiviral transduction of epitope-tagged TDRD7/Tdrd7, IB: Immunoblot.
(PDF)
