# Supplementary figures and images for: A new mechanism of interferon’s antiviral action: Induction of autophagy, essential for paramyxovirus replication, is inhibited by the interferon stimulated gene, TDRD7
Source: PLoS Pathog. 2018 Jan 30;14(1):e1006877. doi: 10.1371/journal.ppat.1006877 (PMC5806901; doi:10.1371/journal.ppat.1006877)

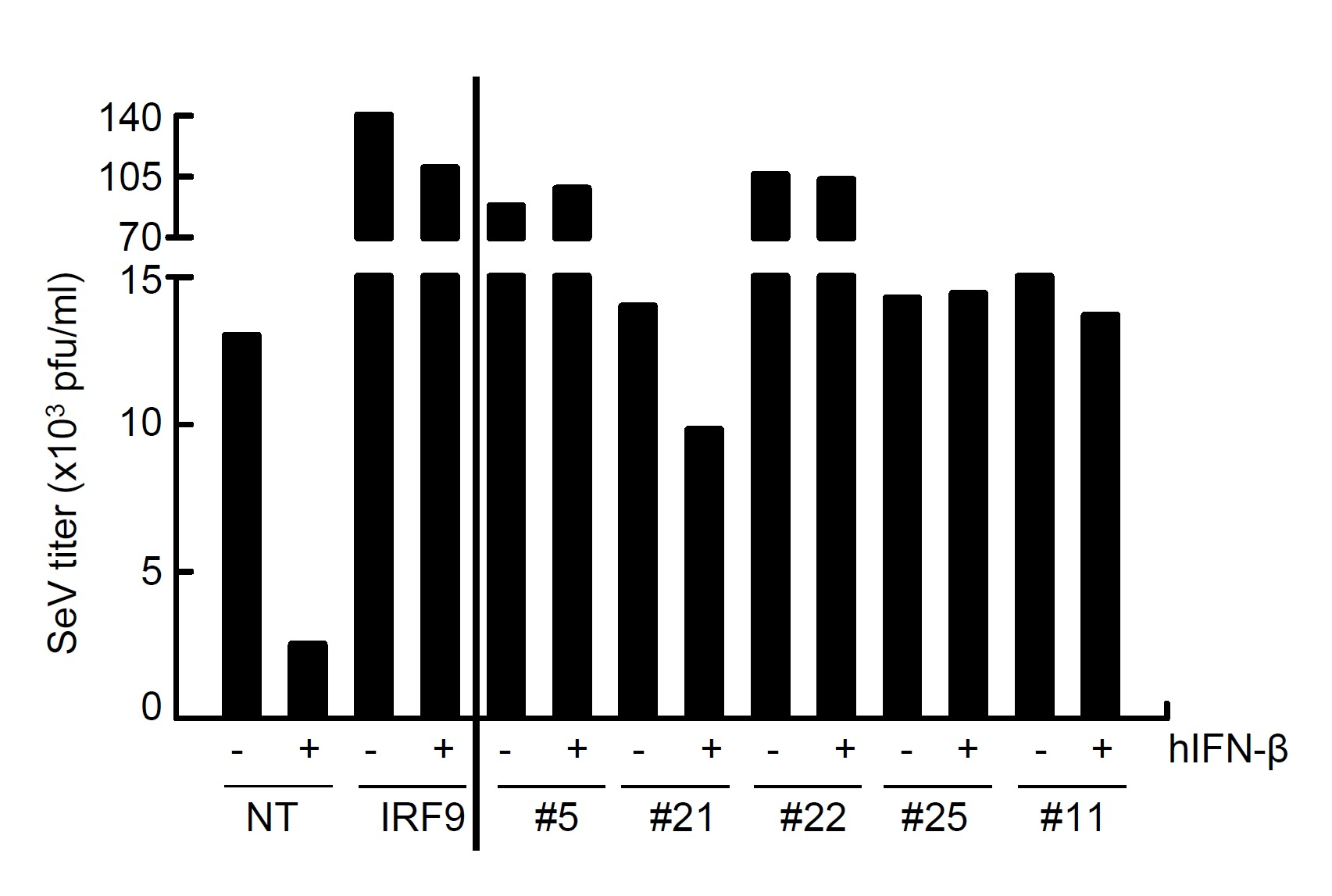

Supplement: S1 Fig — HeLa cells expressing non-targeting (NT), IRF9 or the ISG-specific shRNA (numbers are indicated in Fig 2B), were left untreated or IFN-β-treated and SeV titers were determined at 24 hpi in the culture supernatants. (TIF) [file ppat.1006877.s001.tif]

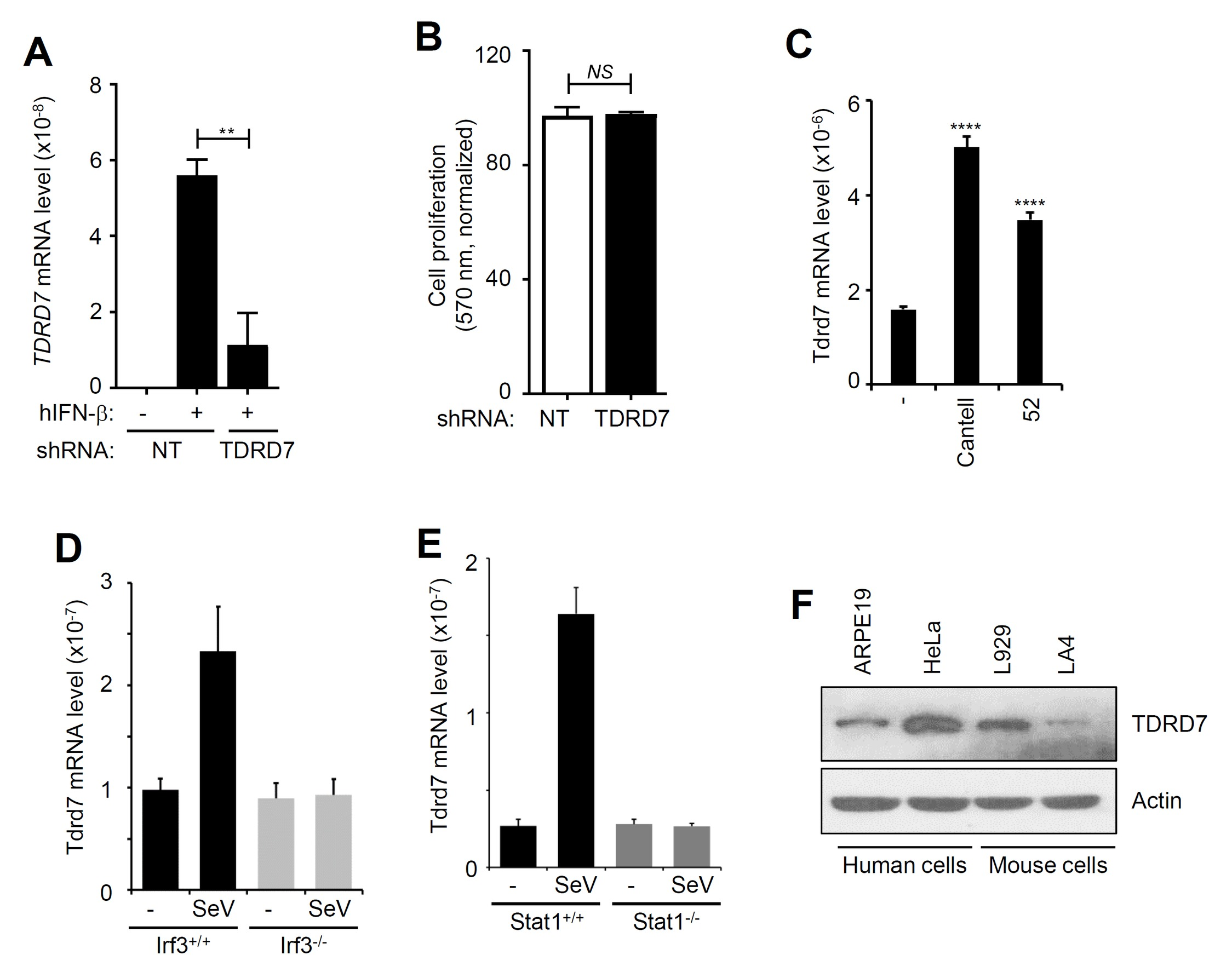

Supplement: S2 Fig — (A) Stable knockdown of TDRD7 in IFN-treated HeLa cells were analyzed by qRT-PCR. (B) Cell proliferation was analyzed in TDRD7 knockdown HeLa cells by MTT assay. (C) Primary BMDMs from Wt C57BL/6 mice were infected with SeV (Cantell and 52 strains) and Tdrd7 mRNA levels were analyzed by qRT-PCR 8 hpi. (D, E) MEFs from the indicated genotypes were infected with SeV at moi:10 and Tdrd7 mRNA levels were analyzed by qRT-PCR. (F) Endogenous TDRD7 protein expression in various human and mouse cells was analyzed by immunoblot. NT, non-targeting. (TIF) [file ppat.1006877.s002.tif]

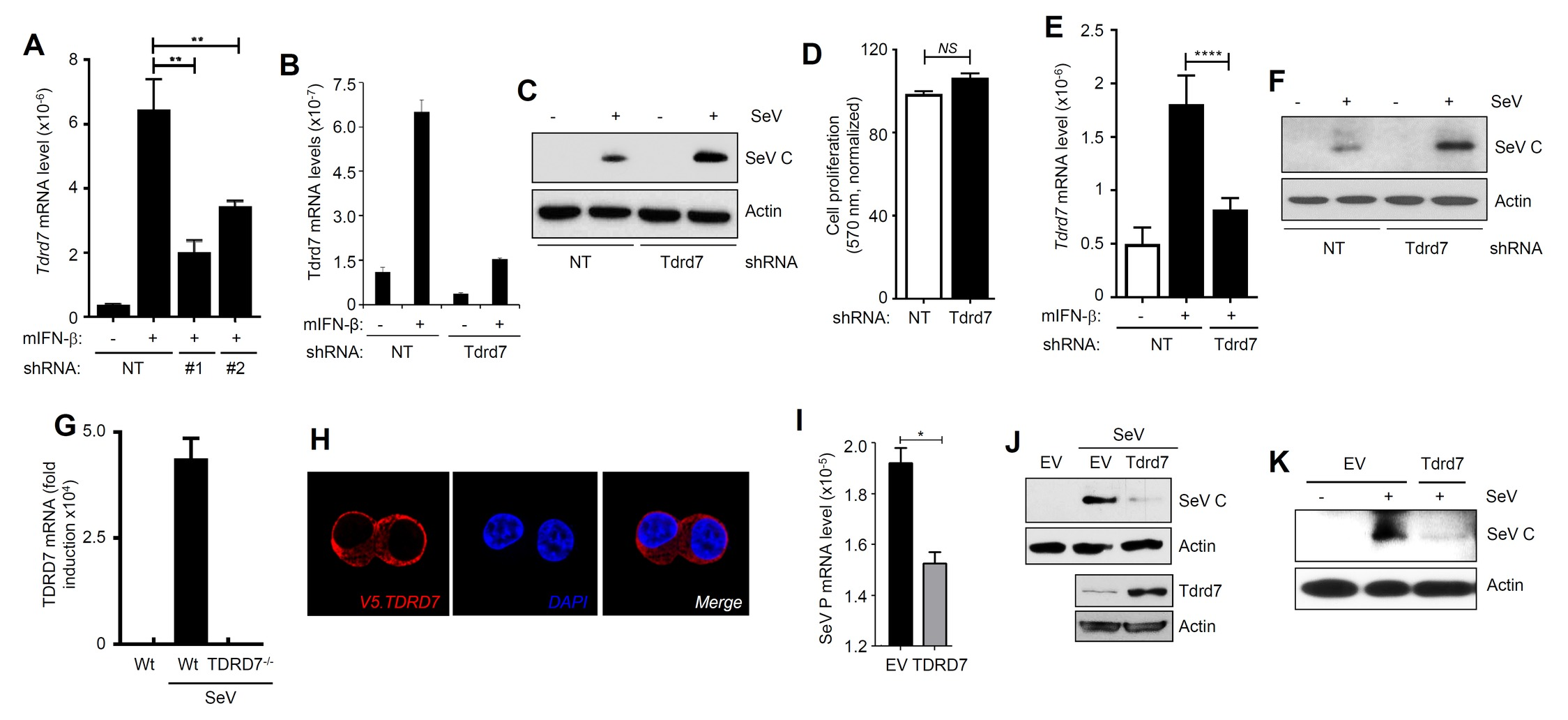

Supplement: S3 Fig — (A) Stable knockdown of Tdrd7 in LA4 cells by two different shRNAs (#1 and #2) was analyzed by qRT-PCR. (B) Stable knockdown of Tdrd7 in L929 cells was analyzed by qRT-PCR. (C) L929 cells expressing Tdrd7-specific shRNA were infected with SeV and SeV C levels were analyzed by immunoblot. (D) Cell proliferation was analyzed in Tdrd7 knockdown L929 cells by MTT assay. (E) Stable knockdown of Tdrd7 in IFN-treated MEFs was analyzed by qRT-PCR. (F) MEFs expressing Tdrd7-specific shRNA were infected with SeV and viral protein (SeV C) levels were analyzed by immunoblot. (G) Wt and TDRD7-/- HT1080 cells were mock-infected or infected with SeV and TDRD7 mRNA levels were analyzed by qRT-PCR. (H) HEK293T cells, stably expressing V5.TDRD7, were immuno-stained with anti-V5 antibody and analyzed by confocal microscopy. (I) HEK293T cells, stably expressing V5.TDRD7, were analyzed for SeV P mRNA expression by qRT-PCR after 8h of SeV infection. (J) L929 cells ectopically expressing Tdrd7 (Origene, untagged) were infected with SeV and SeV C levels were analyzed by immunoblot. Lower panel indicates the ectopic expression of Tdrd7. (K) LA4 cells expressing Tdrd7 (Origene, untagged) were infected with SeV and analyzed for SeV C levels by immunoblot. NT, non-targeting, EV, empty vector. (TIF) [file ppat.1006877.s003.tif]

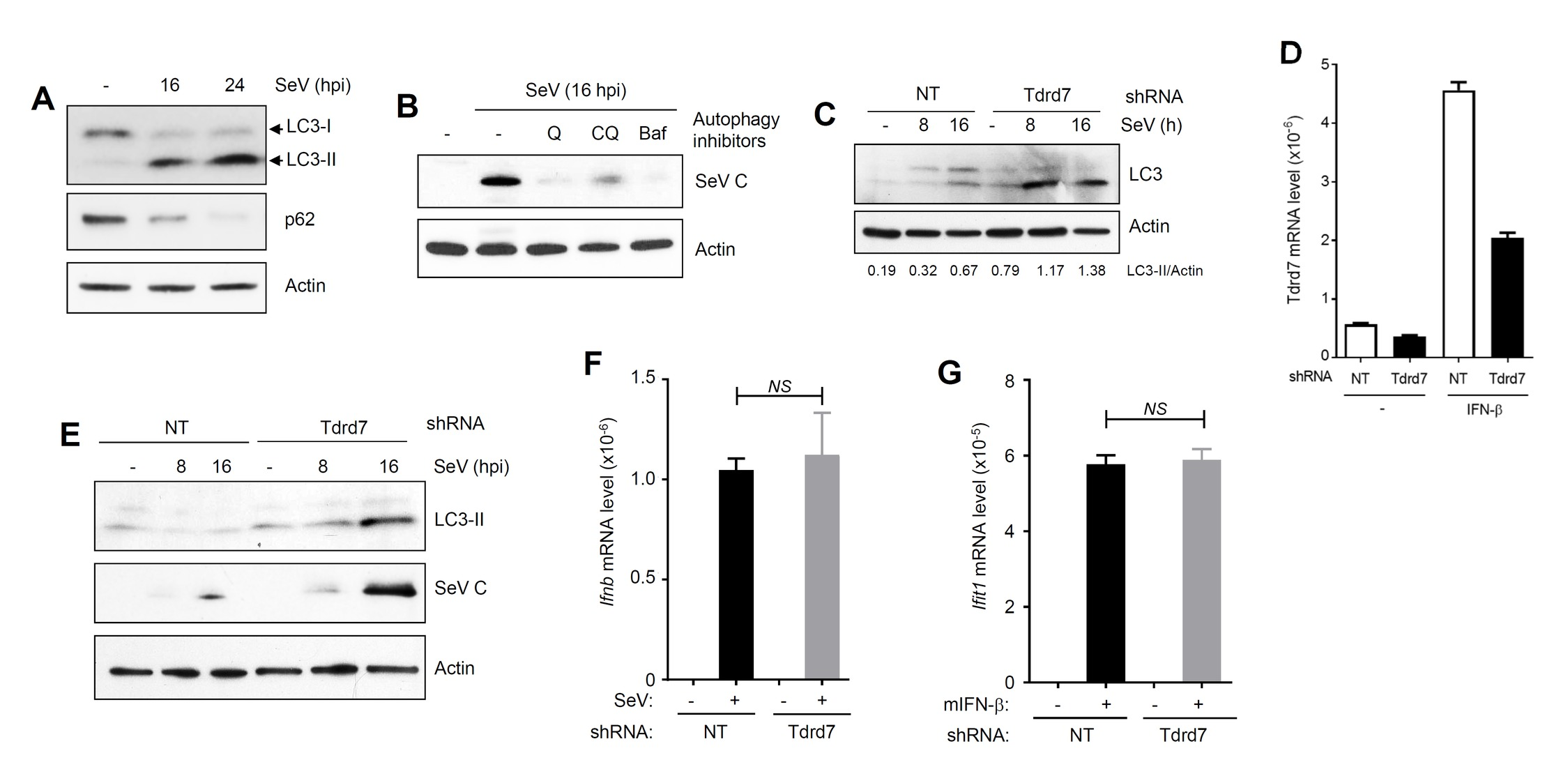

Supplement: S4 Fig — (A) LA4 cells infected with SeV (moi:10) for the indicated time, when LC3-II and p62 levels were analyzed by immunoblot. (B) HT1080 cells were pre-treated with autophagy inhibitors (Q, quinacrine 10μM, CQ, chloroquine 25μM, Baf, Bafilomycin 100 nM) and infected with SeV for 16h, when SeV C levels were analyzed by immunoblot. (C) ARPE19 cells expressing Tdrd7-specific shRNA, were infected with SeV and LC3 levels were analyzed by immunoblot. (D) RAW264.7 cells expressing Tdrd7-specific shRNA, were treated with IFN-β and Tdrd7 mRNA levels were analyzed by qRT-PCR. (E) RAW264.7 cells expressing Tdrd7-specific shRNA were infected with SeV for the indicated time, when LC3-II and SeV C levels were analyzed by immunoblot. (F) Ifnb induction was analyzed in Tdrd7 knockdown L929 cells upon SeV infection by qRT-PCR. (G) Ifit1 induction was analyzed in Tdrd7 knockdown L929 cells upon mIFN-β treatment by qRT-PCR. NT, non-targeting. (TIF) [file ppat.1006877.s004.tif]

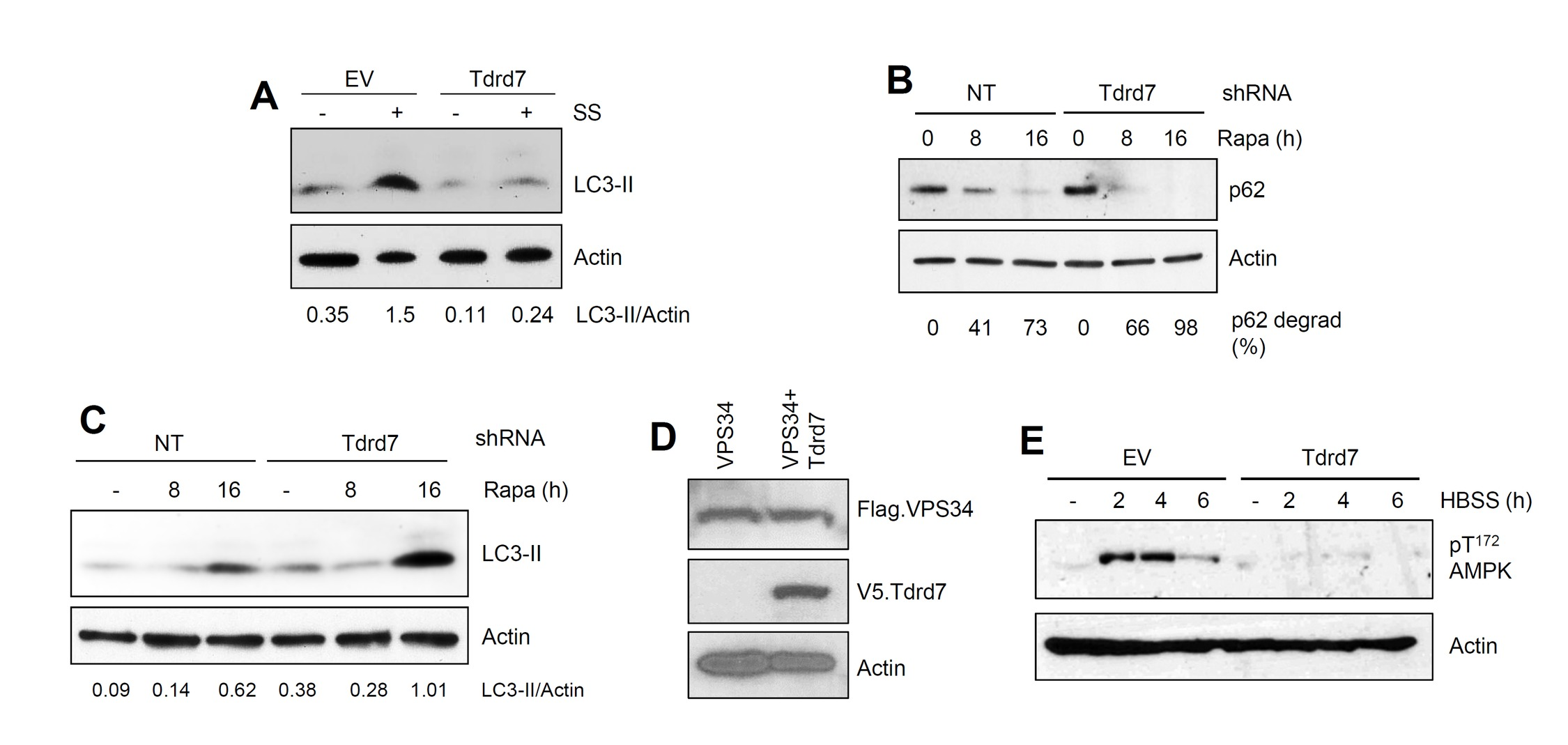

Supplement: S5 Fig — (A) L929 cells stably expressing V5.Tdrd7 were serum-starved (SS) for 16h, when LC3-II levels were analyzed by immunoblot. LC3-II/Actin ratio are shown below the Actin panel. (B, C) RAW264.7 cells expressing Tdrd7-specific shRNA were treated with rapamycin (Rapa) for the indicated time when p62 (B) and LC3-II (C) levels were analyzed by immunoblot. (D) Expression of Flag.VPS34 and V5.Tdrd7 was analyzed in transfected L929 cells by immunoblot. (E) L929 cells stably expressing V5.Tdrd7 were incubated in HBSS for the indicated time, when pAMPK (on Thr172) levels were analyzed by immunoblot. NT, non-targeting, EV, empty vector. (TIF) [file ppat.1006877.s005.tif]

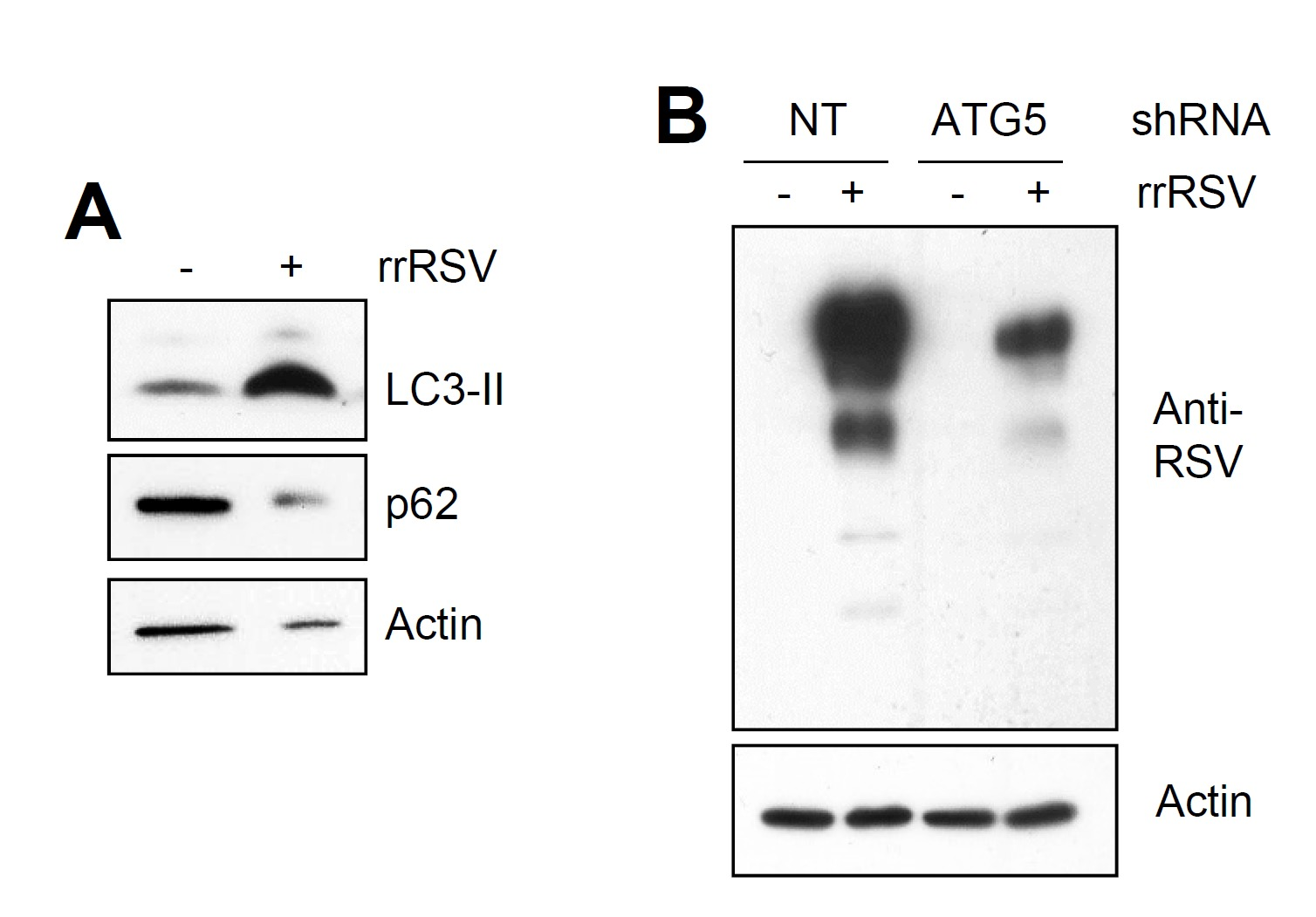

Supplement: S6 Fig — (A) HT1080 cells, infected with rrRSV were analyzed for LC3-II and p62 by immunoblot 24 hpi. (B) HT1080 cells expressing non-targeting (NT) or ATG5-specific shRNA, were infected with rrRSV and analyzed for viral protein expression at 48 hpi. (TIF) [file ppat.1006877.s006.tif]

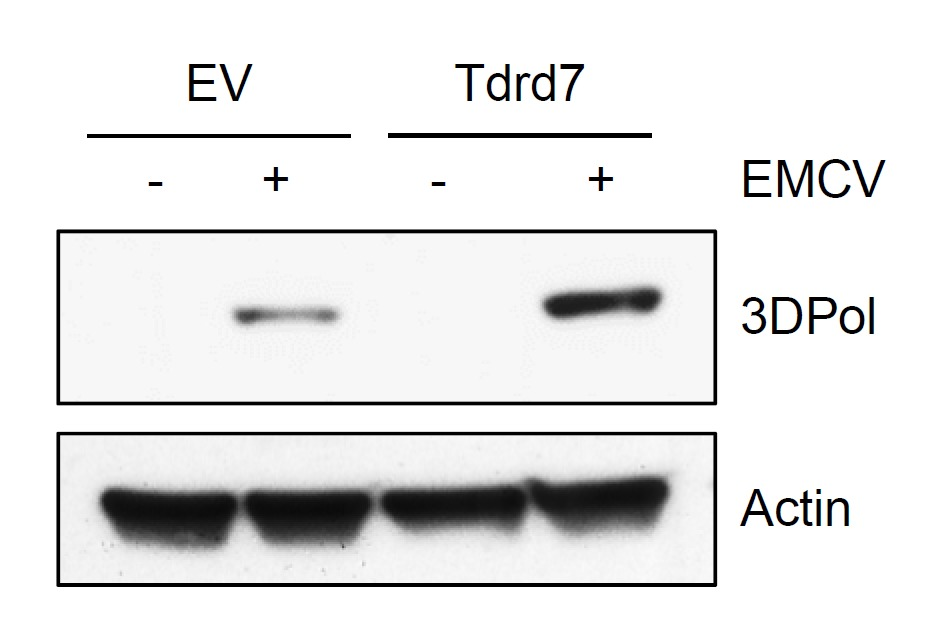

Supplement: S7 Fig — L929 cells ectopically expressing Tdrd7 were infected with EMCV (moi:1) and viral RNA polymerase (3DPol) expression was analyzed by immunoblot after 8 h. EV, empty vector. (TIF) [file ppat.1006877.s007.tif]
