# Supplementary material for: A new mechanism of interferon’s antiviral action: Induction of autophagy, essential for paramyxovirus replication, is inhibited by the interferon stimulated gene, TDRD7
Source: PLoS Pathog. 2018 Jan 30;14(1):e1006877. doi: 10.1371/journal.ppat.1006877 (PMC5806901; doi:10.1371/journal.ppat.1006877)
Supplement: S1 Table — The percent SeV infectivity for each ISG shRNA (as shown in Fig 1D) was used to calculate the z-scores and normalized to that of IRF9 shRNA. The normalized z-scores of each of the ISG shRNA in the library are shown in the table. (PDF) [file ppat.1006877.s008.pdf]

ISGs	z-score
GPR126	3.631111111
IL13RA1	3.386479592
ADAR	3.278290598
KBTBD8	3.263589744
ZNFX1	3.06075
RGS22	2.925918367
SC4MOL	2.849
IL8	2.82975
NMI	2.826581633
UGT1A1	2.695
TRIM69	2.631452991
EMR1	2.602051282
CASP1	2.484444444
LAMP3	2.474387755
EIF2AK2	2.337435897
NAMPT	2.308034188
IL4I1	2.249230769
DDX58	2.19042735
ATL1	2.102222222
GBP5	2.0405
NPIP	2.0405
TDRD7	2.016944444
PAK3	2.014017094
GPR126	1.940512821
PNPT1	1.90575
DHX58	1.86725
PPM1K	1.864722222
SERPINB7	1.85127551
TNFSF10	1.819055556
IL8	1.79025
AGXT	1.75175
IL18	1.71325
ACSL4	1.652602041
AURKB	1.636388889
PAK3	1.631794872
DDX3X	1.59775
SCGB2A1	1.491777778
PPP2R2A	1.48225
CTSS	1.47008547
ASCC3	1.463
CXCR4	1.455384615
CHAC1	1.435867347
WDFY1	1.430888889
KRT1	1.4245
TLR3	1.40877551
RPL41	1.408055556
SYNJ1	1.36675
SP110	1.323076923

UBD	1.323076923
IFIH1	1.293888889
Pmp22	1.293675214
IFIT3	1.265
DDX58	1.255255102
Acsl4	1.237193878
USP18	1.234871795
STAT1	1.226851852
TRIM34	1.205470085
FAM106A	1.201071429
CXCL1	1.1935
CCNG1	1.19
LMO2	1.155
OAS1	1.149277778
CD38	1.138518519
C2orf30	1.13
MOV10	1.058461538
UBE2L6	1.043760684
APOL1	1.025
APOD	1.011428571
SP100	1.004666667
NT5C3	0.984375
PPM1K	0.98175
PNPT1	0.98
STAT2	0.966611111
IFIT2	0.945
TNFSF13B	0.921484375
ANKS1B	0.915
CXCR4	0.911452991
USP18	0.904928571
TAP2	0.90475
IL13RA1	0.8905
HTR1D	0.8855
CD47	0.846357143
TOR3A	0.822
MOV10	0.821307506
ICAM1	0.812755102
ADAR	0.808547009
TNFSF13B	0.8085
GBP3	0.791555556
GBP5	0.776632653
TRIM6	0.77537037
OGFR	0.755740741
IRF9	0.755
IFIH1	0.75075
GLI3	0.75075
ZEB2	0.75
TRIM34	0.744309927
SLC15A3	0.744309927

HLA-DQA1	0.74375
IFNGR1	0.74
TNFAIP6	0.7315
OAS2	0.729214286
NPC2	0.716481481
TAP2	0.71225
TRIM21	0.705811138
TRIM34	0.690940171
EIF2AK2	0.685
LGALS3BP	0.680145278
KRT1	0.678125
LOC339047	0.677295918
TMOD3	0.677295918
ICAM3	0.677222222
CXCL10	0.661538462
SYNJ1	0.655
TNFSF10	0.6545
B2M	0.648046875
IRF9	0.647214286
PARP12	0.647214286
LAP3	0.645
ADAR	0.632136752
UBD	0.628148148
IL18	0.615
NP	0.6125
SP110	0.586055556
SC4MOL	0.578444444
GTPBP2	0.577959184
NPM3	0.57748184
HTR1D	0.575
TTC39B	0.569259259
IFITM2	0.563222222
FCGR1A	0.559444444
SAMD9	0.541785714
ZC3HAV1	0.54
MOV10	0.538983051
CCNG1	0.533203125
JAK2	0.527734375
TRIM34	0.526150121
PSME2	0.52377551
SP100	0.516796875
DDX58	0.514529915
GPR126	0.513317191
FAM106A	0.5125
IFIT2	0.51
C2orf30	0.505
STAT2	0.495
SEMA6D	0.48671875
ATF3	0.486142857

CCL4	0.486142857
STAT3	0.485128205
LGALS3BP	0.485128205
TTC39B	0.485
UBE2L6	0.480925926
RGS22	0.4795
REEP3	0.473046875
STAT3	0.47042735
DDX3X	0.47042735
ZNF148	0.468571429
MAP3K8	0.465
CTTNBP2NL	0.459785714
TRIM69	0.455726496
IFI16	0.451171875
STAT1	0.45
IL18	0.448071429
MX2	0.440234375
APOD	0.44
CD38	0.435
SP110	0.427571429
PAK3	0.423828125
DOCK4	0.4235
C2orf64	0.423486683
SOCS1	0.422037037
JAK2	0.418611111
C2, CFB	0.412222222
BST2	0.407421875
FCGR1A	0.406377551
GBP3	0.404142857
LPGAT1	0.397346939
AKT3	0.395
SNX5	0.392592593
STAT3	0.384987893
FAM3C	0.382222222
NAMPT	0.380714286
TAP2	0.379285714
FKBP1B	0.379285714
AIDA	0.375
CXCL11	0.372944444
REC8	0.363671875
TNFSF13B	0.360214286
IFI44L	0.357722222
GMPR	0.351428571
SPTBN4	0.343163265
INDO	0.333857143
PLSCR1	0.328
AIDA	0.327277778
REEP3	0.323888889
ADAR	0.323418803

PLSCR1	0.322142857
CXCR4	0.320823245
AKAP8	0.320823245
MOV10	0.316285714
PML	0.316071429
NAMPT	0.31171875
C2orf30	0.31
AKAP8	0.308717949
RARRES3	0.296833333
SERPING1	0.295785714
NMI	0.294444444
CD38	0.278214286
CASP1	0.275
ATL3	0.272357143
IFRD1	0.270918367
NT5C3	0.269428571
PKLR	0.261887755
LPGAT1	0.260642857
C19orf66	0.257714286
PML	0.256658596
ATL1	0.254785714
ACSL4	0.251857143
DDX3X	0.25025
CD47	0.245
HTR1D	0.243825666
HERC6	0.235944444
LGALS3BP	0.234795918
PAK3	0.230992736
EIF2AK2	0.222571429
GNB4	0.219642857
PMP22	0.216015625
C2orf30	0.215925926
BTC	0.21175
EXOC4	0.197888889
CXCL10	0.195
EMR1	0.193285714
EXOC4	0.193285714
EMR1	0.192493947
LOC727996	0.192493947
C2orf64	0.192493947
MAP3K8	0.187428571
TXNDC17	0.178642857
IFIT5	0.176666667
KLK8	0.175055556
SEC14L3	0.169857143
TMOD3	0.166928571
HMG1L6	0.165
NFS1	0.165
IRF1	0.164

PSME2	0.16255102
LGALS9	0.161071429
OSBPL9	0.161071429
TRIM22	0.159833333
LOC727996	0.153995157
HMG1L6	0.152222222
CXCL1	0.147222222
DDX3X	0.137407407
DTX3L	0.13475
TRIM34	0.128329298
OAS2	0.125928571
TCIRG1	0.125
HRASLS2	0.12
SAMHD1	0.12
GPR126	0.117606838
REC8	0.1155
TRIM6	0.115496368
TRIM34	0.115496368
SLC1A3	0.115496368
GNB4	0.107962963
DDX3Y	0.107962963
MOV10	0.102905983
BIRC3	0.1
IFI6	0.099571429
EPC1	0.098944444
HLA-B	0.098944444
ACSL4	0.09
SOCS1	0.08203125
XAF1	0.082
GNB4	0.08127551
AIDA	0.08
IFNGR1	0.078518519
DEFA1	0.07
CCNG1	0.068703704
SCGB2A1	0.068359375
LAMP3	0.067357143
CCNG1	0.065625
NPM3	0.064164649
AKAP8	0.062890625
TRIM34	0.058803419
IFI6	0.05775
SERPING1	0.05775
EIF2AK2	0.051953125
AKAP8	0.051331719
GMPR	0.049074074
GBP3	0.046857143
RNASE6	0.045666667
TRIM34	0.039259259
SERPINB7	0.03828125

NUB1	0.036122449
PSMB9	0.030078125
DEFA1	0.02734375
UBE2L6	0.02734375
AURKB	0.026357143
SERPINB7	0.026357143
IFI44L	0.021875
TRIM69	0.0205
IL8	0.01962963
SEC14L3	0.01962963
RSAD2	0.017571429
FKBP1B	0.01640625
HRASLS2	0.015222222
EMR1	0.015
SLC15A3	0.01283293
SP100	0.0109375
OSBPL9	0.008785714
MSMB	0.008785714
GBP2	0.007611111
B2M	0
PPP2R2A	0
SLC45A2	0
ANKFY1	0
DOCK4	0
GBP4	0
EIF2AK2	0
NMI	0
DTX3L	0
HSD17B14	0
APOL1	0
UBA7	0
HERC5	0
MX1	0
ATL3	0
GPR177	0
C2orf30	0
FAM46A	0
ACSL4	0
HERC5	0
IL6	0
RGS22	0
LOC100132540	0
SLAMF7	0
SAMD9	0
DDX58	0
DDX58	0
HIST2H2BE	0
ADAR	0
TRIM14	0

HSD17B14	0
OASL	0
B2M	0
SLC16A4	0
GTPBP2	0
NAMPT	0
IRF8	0
GBP5	0
SAMHD1	0
MAP3K8	0
SYNJ1	0
ZNF384	0
CYP26A1	0
IL8	0
LYPLA1	0
LOC100132540	0
GNB4	0
OSBPL9	0
PARP12	0
TRIM22	0
HLA-F	0
IRF1	0
DHX58	0
PLSCR2	0
KBTBD8	0
IFITM1	0
AKT3	0
ISG15	0
IFRG15	0
LSS	0
HERC6	0
GBP1	0
NFS1	0
SEC14L2	0
STAT3	0
SLC25A28	0
NMI	0
VISA	0
SLC16A4	0
CCNG1	0
PARP9	0
HERC5	0
IFRD1	0
SLC25A28	0
GCH1	0
PARP14	0
ICAM1	0
IFIH1	0
ASCC3	0

NP	0
SAMD9L	0
IFI44	0
GPR177	0
FAM3C	0
ASCC3	0
CCNG1	0
PLSCR1	0
EIF2AK2	0
UBD	0
DDX3X	0
ICAM1	0
SAMD9L	0
MOV10	0
PAK3	0
MOV10	0
MOV10	0
LGALS3BP	0
TRIM21	0
EMR1	0
MOV10	0
TRIM21	0
IL4I1	0
GPR126	0
CXCR4	0
HTR1D	0
ADAR	0
MX1	-0.002734375
IFITM1	-0.002734375
GLI3	-0.005
ATCAY	-0.008785714
RGS22	-0.009030612
TRIM14	-0.011714286
TRIM6	-0.013671875
AKT3	-0.015222222
IRF3	-0.017571429
ANKS1B	-0.018061224
CXCR4	-0.01962963
WDFY1	-0.01962963
ISG20	-0.02
WDFY1	-0.02
SLC45A2	-0.021875
ZC3HAV1	-0.027091837
LPGAT1	-0.027091837
GBP1	-0.029444444
BIRC3	-0.030444444
ANKS1B	-0.035
PARP9	-0.036122449
CCDC50	-0.041015625

C1S	-0.04375
LOC339047	-0.045
LOC100132540	-0.045
LMO2	-0.045153061
ATL1	-0.046857143
CASP1	-0.049074074
IFNGR1	-0.054183673
NLRC5	-0.055642857
DDX24	-0.05775
SERPINB7	-0.060888889
KRT1	-0.0615
TRIM34	-0.064164649
IL6	-0.065
UBA7	-0.065
LMO2	-0.068703704
SAMHD1	-0.072244898
GPR126	-0.073504274
IL4I1	-0.073504274
AIDA	-0.073828125
RNASE6	-0.076111111
TRIM34	-0.077
TRIM25	-0.079296875
ASCC3	-0.090234375
RTP4	-0.090785714
GBP2	-0.090785714
CEACAM1	-0.096642857
LY6E	-0.098148148
PPM1K	-0.098148148
LGALS9	-0.098148148
SEMA6D	-0.098148148
ZNF148	-0.0984375
SC4MOL	-0.099336735
SLC1A3	-0.102663438
NFS1	-0.105428571
LPGAT1	-0.106640625
APOL6	-0.107962963
LSS	-0.108367347
SCGB2A1	-0.108367347
DOCK4	-0.11
HLA-DQA1	-0.111285714
ZNF384	-0.115
AGT	-0.117578125
KBTBD8	-0.117606838
MYO1C	-0.121777778
REC8	-0.128515625
IFI35	-0.128857143
GTPBP2	-0.128857143
ATL3	-0.13
EMR1	-0.132307692

TLR3	-0.133984375
DDX24	-0.13475
UGT1A1	-0.135
SOCS1	-0.135459184
IFRD1	-0.13671875
CXCR4	-0.137642857
NAMPT	-0.1421875
CA13	-0.1435
VISA	-0.144921875
IL1RN	-0.158142857
GMPR	-0.161328125
IFI27	-0.161328125
XDH	-0.16255102
PARP9	-0.16255102
IFRG15	-0.164
KLK10	-0.1640625
SLC16A4	-0.166851852
IL1RN	-0.166851852
C19orf66	-0.167444444
LOC100131940	-0.167444444
DDX24	-0.167444444
BATF2	-0.175055556
ANP32B	-0.18046875
IFIT3	-0.18046875
AKT3	-0.180612245
DTX3L	-0.182666667
CA13	-0.1845
PMP22	-0.1859375
SERPINB7	-0.186481481
KRT1	-0.186481481
IL18	-0.189642857
PSME2	-0.189642857
AKT3	-0.190357143
EMR1	-0.191111111
HTR1D	-0.191111111
DTX3L	-0.19140625
GOSR1	-0.199609375
C19orf66	-0.2055
LPGAT1	-0.205811966
TRIM69	-0.205811966
MAFK	-0.207704082
FCGR1A	-0.216734694
PLSCR1	-0.2296875
TRIM6	-0.235
LOC727996	-0.235213675
AKAP8	-0.235213675
PLSCR2	-0.243555556
GBP4	-0.243555556
C2orf64	-0.243825666

CCL4	-0.243826531
EIF2AK2	-0.248828125
PPP2R2A	-0.25025
AIM1	-0.2515625
SLC25A28	-0.252857143
NLRC5	-0.254296875
NPM3	-0.256658596
MAFK	-0.258777778
AGT	-0.259765625
SP140	-0.26
FAM46A	-0.261887755
ATCAY	-0.261887755
CTTNBP2NL	-0.266388889
APOL1	-0.26796875
PLA1A	-0.26796875
IL4I1	-0.270918367
FAM3C	-0.272357143
EIF2AK2	-0.274
CTSS	-0.276171875
TRIM6	-0.27890625
TMOD3	-0.281611111
TGM2	-0.281611111
TMEM146	-0.281640625
CTTNBP2NL	-0.28462963
OAS1	-0.287109375
TRIM21	-0.28875
KLK8	-0.289222222
B2M	-0.295
ZC3HAV1	-0.295785714
CCDC50	-0.296833333
MAFK	-0.296833333
PARP9	-0.30625
LGALS9	-0.307040816
RSAD2	-0.308984375
IRF1	-0.308984375
MYD88	-0.31
KRT1	-0.312055556
PLA1A	-0.312055556
DAXX	-0.312055556
CCL5	-0.312265625
TRIM6	-0.312539063
NPC2	-0.313357143
NFIL3	-0.314074074
CXCL1	-0.314074074
INDO	-0.314074074
SP140	-0.314074074
LAMP3	-0.316071429
FTL	-0.316285714
SLC45A2	-0.316285714

CDKN1A	-0.319648438
ATCAY	-0.325
TNFSF13B	-0.325102041
IFIT5	-0.325755556
APOL6	-0.331844444
IRF9	-0.333867188
TAPBP	-0.334132653
GCH1	-0.334132653
BLMH	-0.3390625
HLA-A	-0.339609375
STAT1	-0.339714286
WDFY1	-0.343163265
GBP2	-0.345
LOC23117	-0.3485
TRIM22	-0.353333333
CHAC1	-0.353333333
HCP5	-0.359023438
SLC15A3	-0.359322034
CXCL11	-0.360005556
IFITM2	-0.36122449
ATF3	-0.36122449
AURKB	-0.361757813
ZNFX1	-0.370255102
SEC14L2	-0.37515625
EIF2AK2	-0.387405556
ATL3	-0.388316327
GPR126	-0.3915
HIST2H2BE	-0.392592593
NFIL3	-0.395357143
KBTBD8	-0.396923077
TRIM14	-0.397304688
HRASLS2	-0.401866667
FAM129A	-0.409477778
IRF1	-0.421714286
BIRC3	-0.424438776
ANKS1B	-0.430788889
SYNJ1	-0.431851852
SLC15A3	-0.436319613
BLMH	-0.439285714
TRIM25	-0.441025641
ACSL4	-0.441666667
NFS1	-0.442214286
PSMB8	-0.442214286
IFIT1	-0.4525
GBP4	-0.453085938
IRF8	-0.455
GPR126	-0.456666667
WIPF1	-0.459785714
B2M	-0.460561224

APOD	-0.461
APOL1	-0.462
PELI1	-0.462755556
IRF7	-0.468571429
CASP12	-0.471111111
TRIM34	-0.474818402
HTR1D	-0.474818402
TRIM22	-0.480925926
IFIT2	-0.483305556
CXCR4	-0.487651332
CEACAM1	-0.490740741
FTL	-0.496683673
LPGAT1	-0.496683673
IRF7	-0.499288889
TCIRG1	-0.499296875
NPC2	-0.51037037
ACRC	-0.51037037
GMPR	-0.512988889
ICAM1	-0.51375
IFI16	-0.514744898
BTC	-0.51975
PSMB9	-0.520625
CDKN1A	-0.522883333
MYD88	-0.523
CXCR4	-0.526150121
GPR177	-0.5265
MAFK	-0.535928571
NUB1	-0.538105556
TMEM140	-0.539814815
SLAMF7	-0.544714286
DDX58	-0.550867347
UBD	-0.56464891
IFIT2	-0.564744444
IL13RA1	-0.564744444
SOCS1	-0.568928571
SLC15A3	-0.57748184
ATP6V0A1	-0.5775
AKAP8	-0.579205556
IFRG15	-0.58515625
MOV10	-0.59031477
SLC15A3	-0.59031477
FCGR1A	-0.5905
GCH1	-0.592
GPR126	-0.596020408
LTF	-0.596020408
GBP4	-0.596020408
CCL5	-0.6155
TRIM6	-0.618783333
BIRC3	-0.621827778

OGFR	-0.623112245
FKBP1B	-0.623112245
CA13	-0.623785714
HERC6	-0.628
ZEB2	-0.6285
ATP6V0A1	-0.629438889
IL4I1	-0.632136752
CDKN1A	-0.637811111
IFI44L	-0.646944444
HCN3, PKLR	-0.647214286
BTC	-0.658928571
MOV10	-0.667714286
VISA	-0.670642857
CTTNBP2NL	-0.672822222
GCH1	-0.677295918
APOBEC1	-0.67815
PLSCR1	-0.68
APOL6	-0.682357143
AKT3	-0.684
CTSS	-0.6845
ASCC3	-0.6855
LOC728467	-0.686326531
PLA1A	-0.686522222
IRF1	-0.687037037
DAXX	-0.6885
PMP22	-0.6895
STAT2	-0.693
IFI44L	-0.693
APOL2	-0.6945
TRIM6	-0.706666667
ANKFY1	-0.713418367
TRIM34	-0.718644068
TRIM21	-0.72034188
AKAP8	-0.722
PSME2	-0.7261
HSD17B14	-0.726285714
SOCS1	-0.728383333
LOC100132247	-0.729
DDX24	-0.737
PSMB9	-0.740510204
IFIT1	-0.7435
TRIM25	-0.744309927
IRF1	-0.744309927
TMEM173	-0.744366667
GBP4	-0.7465
PARP14	-0.747
TRIM69	-0.7485
FAM129A	-0.749540816
TRIM25	-0.74974359

TRIM6	-0.74974359
STAT1	-0.751
IFI27L2	-0.753
CCDC50	-0.758571429
HERC6	-0.776632653
C2, CFB	-0.776632653
CDKN1A	-0.776632653
CEACAM1	-0.779
DDX24	-0.780138889
TRIM21	-0.785663265
STAT3	-0.794
RGS22	-0.795
ATL3	-0.80372449
SEMA6D	-0.80372449
AKAP8	-0.821307506
LOC652671	-0.823247863
KBTBD8	-0.82775
TRIM6	-0.839846939
CXCR4	-0.839846939
DHX58	-0.848877551
IFRD1	-0.848877551
GOSR1	-0.858533333
SAMD9	-0.858533333
WIPF1	-0.875935714
PARP14	-0.878322222
MSMB	-0.886771429
GBP1	-0.888821429
KBTBD8	-0.896752137
ZNFX1	-0.90475
ANKFY1	-0.90475
JAK2	-0.912091837
CXCL1	-0.924
AKAP8	-0.99965812
JAK2	-1.029489796
PNPT1	-1.073166667
CEACAM1	-1.073166667
TTC39B	-1.073166667
SNX5	-1.073166667
IL6	-1.078
AKAP8	-1.087863248
OAS2	-1.09725
XDH	-1.17425
LAP3	-1.192040816
ACSL4	-1.1935
TLR3	-1.1935
HSH2D	-1.21275
DDX3X	-1.22017094
MX1	-1.264273504
LGALS3BP	-1.278974359

TRIM21	-1.293675214
CHAC1	-1.309
PMP22	-1.345561224
STAT3	-1.47008547
IFI27	-1.481055556
GBP5	-1.48225
CYP26A1	-1.67475
NFS1	-1.67475
LOC727996	-1.72
ZNF384	-1.98275
NUB1	-2.058119658
PML	-2.234529915
PMP22	-2.25225
PARP9	-2.31
ACSL4	-2.36775
ANKS1B	-2.375185185
TRIM34	-2.396239316
PAK3	-2.425641026
CASP12	-2.59875
TRIM21	-2.69025641
IL4I1, NUP62	-2.71965812
PSMB10	-2.772
CXCR4	-2.807863248
APOL1	-3.175384615
KBTBD8	-3.444410256
UBD	-3.495863248
ANP32B	-3.59975
DDX3X	-3.72225641
PAK3	-4.545504274
PARP12	-6.468
