# Supplementary material for: A new mechanism of interferon’s antiviral action: Induction of autophagy, essential for paramyxovirus replication, is inhibited by the interferon stimulated gene, TDRD7
Source: PLoS Pathog. 2018 Jan 30;14(1):e1006877. doi: 10.1371/journal.ppat.1006877 (PMC5806901; doi:10.1371/journal.ppat.1006877)
Supplement: S2 Table — The table summarizes the properties of various human and mouse cell types, used in the study, with respect to their levels of Tdrd7 mRNA and protein expression, antiviral activity and biochemical mechanism. KD, knockdown by lentiviral transduction of shRNA plasmids, KO, knockout by CRISPR/Cas9, ectopic expression by lentiviral transduction of epitope-tagged TDRD7/Tdrd7, IB: Immunoblot. (PDF) [file ppat.1006877.s009.pdf]

## Table S2: Evaluation of antiviral activity of TDRD7 in various human and mouse cell types

| Cell line | Cell type | Tdrd7 expression | Approach | Virus | Used for |
|---|---|---|---|---|---|
| HeLa | Human epithelial | Yes, tested by qRT-PCR and IB, higher protein expression compared to ARPE19 (by IB) | KD | SeV<br>HPIV3<br>RSV | High throughput screen and virus replication |
| ARPE19 | Human retinal epithelial | Yes, tested by IB, low protein expression compared to HeLa (by IB) | KD | SeV | Virus replication and autophagy mechanism |
| HT1080 | Human fibroblasts | Yes, tested by IB | KO | SeV | Virus replication and autophagy mechanism |
| HEK293T | Human epithelial | Not tested | Ectopic | SeV | Virus replication and autophagy mechanism |
| L929 | Mouse fibroblasts | Yes, tested by qRT-PCR and IB, higher protein expression compared to LA4 (by IB) | KD (shRNA#1) and ectopic | SeV<br>EMCV | Virus replication and autophagy mechanism |
| LA4 | Mouse lung epithelial | Yes, tested by qRT-PCR and IB, low protein expression compared L929 (by IB) | KD (shRNA#1, #2) and ectopic | SeV | Virus replication |
| RAW264.7 | Mouse macrophage | Yes, tested by qRT-PCR | KD (shRNA#1) | SeV | Virus replication and autophagy mechanism |
| MEF | Mouse embryonic fibroblasts | Yes, tested by qRT-PCR | KD (shRNA#1) | SeV | Virus replication |
